# Lost, gained, and regained functional and phylogenetic diversity of European mammals since 8000 years ago

**DOI:** 10.1111/gcb.16316

**Published:** 2022-07-07

**Authors:** Jack H. Hatfield, Katie E. Davis, Chris D. Thomas

**Affiliations:** ^1^ Leverhulme Centre for Anthropocene Biodiversity University of York York UK; ^2^ Department of Biology University of York York UK

**Keywords:** biogeography, biological invasion, colonization, conservation, extinction, extirpation, functional diversity, phylogenetic diversity, rewilding

## Abstract

Mammals have experienced high levels of human‐mediated extirpations but have also been widely introduced to new locations, and some have recovered from historic persecution. Both of these processes—losses and gains—have resulted in concern about functional losses and changes in ecological communities as new ecological states develop. The question of whether species turnover inevitably leads to declines in functional and phylogenetic diversity depends, however, on the traits and phylogenetic distinctiveness of the species that are lost, gained, or regained. Comparing ~8000 years ago with the last century, we show that extirpations and range retractions have indeed reduced the functional and phylogenetic diversity of mammals in most European regions (countries and island groups), but species recoveries and the introduction of non‐native species have increased functional and phylogenetic diversity by equivalent or greater amounts in many regions. Overall, across Europe, species richness increased in 41 regions over the last 8000 years and declined in 1; phylogenetic diversity increased in 33 and declined in 12, while functional diversity results showed 20 increases and 25 decreases. The balance of losses (extirpations) and gains (introductions, range expansions) has, however, led to net increases in functional diversity on many islands, where the original diversity was low, and across most of western Europe. Historically extirpated mega‐ and mesofaunal species have recolonized or been reintroduced to many European regions, contributing to recent functional and phylogenetic diversity recovery. If conservation rewilding projects continue to reintroduce regionally extirpated species and domestic descendants of “extinct” species to provide replacement grazing, browsing, and predation, there is potential to generate net functional and phylogenetic diversity gains (relative to 8000 years ago) in most European regions.

## Graphical abstract

Over the last 8000 years, humans have caused the loss of some European mammal species but there have also been gains associated with species introductions and reintroductions, while some species have re‐expanded into their former distributions after the relaxation of past pressures. Taken together, the resultant variety of lifestyles (ecological or functional diversity) and representation of the tree of life (evolutionary diversity) has increased in some countries and regions, whilst declining in others. If conservation and rewilding initiatives release more species or encourage further expansions, then diversity within regions could in future equal or surpass that of 8000 years ago.

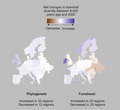

## 1 INTRODUCTION

Extinct species represent phylogenetic losses to the tree of life, while species introductions rearrange the biogeography of evolutionary lineages, hence influencing future ecosystems and evolutionary trajectories indefinitely (Bull & Maron, 2016; Thomas, 2020). The consequences of mammal faunal change are particularly great, given the susceptibility of the megafauna to extinction, and the functional importance of extinct, extirpated, and introduced mammal species within ecosystems (Faurby & Svenning, 2015; Malhi et al., 2016). Not only are these changes of evolutionary significance, but they are of practical relevance to the development of new approaches to conservation, especially rewilding (including reintroductions and facilitated recolonization) and de‐extinction, which most commonly focus on the functional significance of large mammals (Svenning et al., 2016). However, feral populations of deliberately released and escaped mammals also have functional significance. It has been proposed that, in the absence of extinct species, evolutionarily and functionally similar species can be used to restore grazing, browsing, predation, and decomposition processes in ecosystems (Donlan et al., 2006; Falcón & Hansen, 2018). Given that many species of introduced mammals are already widely established around the world, consideration of their functional significance needs to be made alongside reintroduction, rewilding, and de‐extinction projects, which are often justified as a means of restoring historic ecosystem processes. Extirpations and introductions have been subject to separate studies, but their phylogenetic and functional impacts have rarely been considered together, hindering our ability to obtain a balanced picture. Here, we consider both the losses and gains in functional and phylogenetic diversity.

Phylogenetic and functional diversity are important complements to taxonomic diversity (species richness) because they take account of the fact that some species are more distinct than others. Functional diversity focuses on the roles played by species in ecosystems, usually inferred from their traits (Petchey & Gaston, 2006). Phylogenetic diversity considers the branch lengths of species across a phylogenetic tree, with greater branch lengths representing the evolutionary distinctiveness of species (Faith, 1992). The greater the evolutionary diversity of species, the greater the range of ecological roles and traits they are likely to fulfil (including for traits for which direct measurements are not available). These metrics can be used to indicate potential changes to ecological processes, as the losses and gains of distinct species might be expected to have the largest impacts.

We focus on the mammalian fauna of Europe because of its long history of anthropogenic influence, the large impact of mammals on ecosystem structures and processes, and the fact that species extirpations and introductions are relatively well documented. We highlight the last ~8000 years, which has been relatively climatically stable (commencing long enough after the Pleistocene–Holocene climatic transition for climate‐related extirpations and colonizations to be realized) and encompasses many of the large‐scale human impacts in Europe (Turney & Brown, 2007). Over this period, the human population has increased and expanded, brought about extensive land‐use changes, overexploited a range of species, deliberately attempted to exterminate some, transported some species within Europe and imported others from further afield (Crees, Turvey, et al., 2019; Marquer et al., 2017; McClure, 2013). In the light of these changes, we investigate how the taxonomic, functional, and phylogenetic diversity of mammal faunas has changed across the majority of European regions, where regions refer to present‐day countries, historic groupings, islands, or island groups. We consider regions because of the resolution of the available data (zooarchaeological and historical records) and because local mammalian communities are drawn from the species available in regional pools (while the compositions of individual sites vary in relation to multiple factors and change over time). We evaluate the extent to which the diversity reductions resulting from extirpations are offset or even exceeded by gains as a consequence of reintroductions, range expansions, and species introductions. We also investigate the potential gains that could be made by the future re‐establishment or replacement of additional species (e.g., in rewilding projects) and evaluate whether functional and phylogenetic diversity changes are greatest on islands, given the potential loss of endemics and their susceptibility to invasion by new lineages and functional types.

## 2 METHODS

Species lists of terrestrial and freshwater mammals were compiled for each region considered (Table S1). Species present for at least part of the last 8000 years were placed into one of five categories with potential future reintroductions also identified as a sixth category (Tables S2 and S3). For the main analysis, we compare species assemblages at 8000 BP to 2020. Species subgroups (e.g., small mammals) were also used for supplementary analyses (Supporting Information).

### 2.1 Regions

The choice of regional units mainly follows the DAISIE (Delivering Alien Invasive Species Inventories for Europe) dataset (Roy et al., 2019), but we excluded regions that also extend into Asia (e.g., Russia), outlying regions with marine/arctic mammal faunas (The Faroe Islands, Svalbard and Jan Mayen and Iceland) and those with insufficient data (Gibraltar and Monaco). Regions were defined using global administrative boundaries (GADM v3.6, https://gadm.org/; Table S1).

### 2.2 Native species

Lists of extant native mammals were initially created for each region by intersecting boundary polygons with IUCN range maps for terrestrial and freshwater mammals (IUCN, 2021a) in the R package sf (Pebesma, 2018). We included as “native” all species whose presence in a given region was mapped as extant in 2020, using the IUCN maps to classify each species–region combination as native (subsequently identifying reintroductions and excluding non‐native introduced species, below). Some areas suffer from region‐wide data deficiencies; but there were only 53 cases where a species was included but had uncertain origin in a particular region (i.e., it might be native or introduced) based on the map overlaps, of a total of >3000 confirmed species–region combinations. It was possible to subsequently reclassify 51 of the 53 cases as introductions (see below), by checking their origin (native or introduced) against other sources of data, including the IUCN geographic range notes (IUCN, 2021b) and published and online regional/country introduced species lists (directly sourced information was used in preference to that obtained from range map overlaps). There were just 12 instances of uncertain presence across a whole region (IUCN maps showing possible, probable, or uncertain presence), of which 10 related to bat species. Bats are under‐recorded in some regions due to insufficient survey efforts, sparse distributions, and recent taxonomic revisions (IUCN, 2021b). On review against range patterns and notes (IUCN, 2021b), all 12 cases were included as present. To generate 2020 native species lists for each region, we then adjusted lists to take account of different cutoff dates in regional lists and maps (e.g., 1500 https://www.iucnredlist.org/resources/mappingstandards), and to account for regional differences in the definition of native (e.g., to reclassify *Mus musculus* and *Rattus norvegicus* as these are post‐8000 BP introductions to Europe). We also removed species that are not considered terrestrial (e.g., seals).

Native species that were extant in a given region in 2020 were presumed to have also been extant in the same region 8000 BP unless there was direct evidence otherwise (see below). Following these assessments, each terrestrial and freshwater mammal species was placed, for each region, into one of the distribution/origin categories shown in Table S2. All conflicts between data sources were checked and resolved based on strength and weight of evidence (e.g. genetic or zooarchaeological data are more conclusive than expert opinion alone) as well as the known criteria of each source.

### 2.3 Introduced species

Introduced mammal species lists were extracted from two datasets—Global Register of Introduced and Invasive Species (GRIIS; Pagad et al., 2018) and DAISIE (Roy et al., 2019). A number of additional steps were then taken to ensure consistency and reduce any inaccuracies, aiming to include only those species which had reasonable evidence of establishment in a region. All species–region combinations taken from DAISIE and GRIIS were checked against the IUCN Red List (IUCN, 2021b) and the IUCN Invasive Species Specialist Group Global Invasive Species Database (Invasive Species Specialist Group, 2015) between 2020 and 2021. Species–region combinations were only retained if they were listed by two or more sources (either both DAISIE and GRIIS or one of DAISIE/GRIIS and one of the IUCN Red List or Invasive Species Database). Three of the most widespread older introductions (*Mus musculus*, *Rattus rattus*, and *R. norvegicus*) were not listed for Montenegro and Andorra, so we checked their presence against the European Mammal Atlas (Mitchell‐Jones et al., 1999) and IUCN Red List (IUCN, 2021b), adding them to the regional lists where appropriate. Where ambiguity and disagreement around native status exist, we consulted additional literature (Supporting Information, Additional Sources). We only included species recognized by the IUCN (IUCN, 2021b) and did not consider variation below the species level. Although following the IUCN we included the European mouflon (*Ovis aries*), considered an ancient feral population distinct from modern domestic sheep (IUCN, 2021b), other domestic species were not recognized by IUCN (e.g., dogs and cats) and were covered inconsistently by the sources used. The extent to which populations are feral is unclear, considering that domestic dogs and cats interbreed with wolves and wildcats, respectively. Other potentially feral species, such as cattle and horses, are only considered in the section on the potential for rewilding. Note that inclusion of these domestic animals would likely increase the strength of the trends reported (increasing net diversification, especially on islands).

### 2.4 Extirpations and reintroductions

Losses were comprised of species extirpations from a region. We use the term extirpation due to our regional focus, although in some cases, this represented the (global) extinction of a species. Some of these losses were regained when species recolonized or were reintroduced to a region. We use the term reintroduced here to cover both instances, but this should not be taken to imply direct human intervention. To obtain data on these changes, we used advanced searches of the IUCN Red List of Threatened Species (IUCN, 2021b) for mammal species in Europe with status of extinct, possibly extinct, possibly extant, uncertain, or vagrant to identify potential losses and searched for status extant and reintroduced to identify potential reintroductions. We then consulted the geographic range notes for each of these species to identify the evidence of extirpations and reintroductions, consulting additional publications where required (Supporting Information, Additional Sources). In addition, geographic range notes for all species with ranges overlapping the focal region were consulted for any further notes on reintroductions, range expansions, or extirpations. We also used published works that had compiled information on range changes and extirpations. For extirpations and reintroductions, we used the regional species extirpations provided in Crees, Turvey, et al. (2019) with additional details from Crees et al. (2016) and Crees (2013) as well as those listed in Turvey (2009a, 2009b) and Turvey and Fritz (2011). Masini et al.'s (2008) study was used in addition for Sicily as not all sources distinguished it from the Italian peninsula. While the European Holocene faunal record is relatively well documented, it may not be complete (particularly for small mammals, Sommer, 2020; Crees, Collen, & Turvey, 2019), placing a limit on our conclusions regarding historic extirpations.

### 2.5 Future potential reintroductions

We also identified, for each region, species that could potentially be reintroduced (or recolonize) in future. These were species that had been extirpated historically (since 8000 BP) but were (i) still extant in the wild elsewhere (IUCN, 2021b), such as the Eurasian lynx (*Lynx lynx*) which is currently absent from Britain but globally extant or (ii) have similar “surrogates,” for example, certain breeds of domestic animals whose wild ancestor is extinct (Supporting Information). This provides an indication of the diversity that could be accrued if extant species and surrogates (for previously extirpated species) could re‐establish within each region, if the practicalities of doing so could be overcome.

### 2.6 Time periods

We operated an approximate cutoff of 8000 years ago based on agricultural expansion and sea levels (Turney & Brown, 2007). Species that experienced late Pleistocene/early Holocene extirpations (i.e., species with no direct evidence of presence after 8000 BP) were excluded. Similarly, colonizations (and possible introductions) believed to have taken place before 8000 BP were treated as native for the purpose of this analysis. This baseline does not consider earlier human‐caused losses, given that humans and climate change are likely to have combined to cause late Pleistocene/early Holocene extirpations and extinctions in Europe (Koch & Barnosky, 2006; Sandom et al., 2014). An approximate cutoff was used due to high levels of uncertainty in many cases and differences between dating techniques. We used the most recent data available at the time of analysis, meaning that the most recent year considered is 2020. However, time lags in reporting and updating may mean that very recent changes are not able to be considered. In the supplementary analysis, we classified all changes as recent and older using the year 1945, with 1945 or later classed as recent (Supporting Information).

### 2.7 Trait data

Body mass, diet category percentages, foraging strata, and activity period (Smith et al., 2003; Wilman et al., 2014) as well as habitat type (IUCN, 2021b) traits were collected (Table S4). For globally extinct species, additional sources were used (predominantly PHYLACINE 1.2.1; Faurby & Svenning, 2016; Faurby et al., 2018, 2020), averaging across extant relatives where values were not available (Table S5).

### 2.8 Diversity measures

We calculated three measures of mammalian diversity: species richness, phylogenetic diversity, and functional diversity. Species (taxonomic) richness was simply the total number of species in each region for each time period (8000 BP, 1945, supplementary analyses, 2020), rescaled by dividing all species counts by the total number of mammal species (*n* = 228) recorded in Europe in any of the species lists (minimum possible = 0 for no species, maximum = 1 for all species).

Phylogenetic diversity was calculated using Faith's PD (Faith, 1992) in the picante package (Kembel et al., 2010) and 1000 node dated tree samples obtained via VertLife (vertlife.org; Upham et al., 2019), with extinct species added (Table S7). This method summed all branch lengths (the minimum spanning tree for only the species present [pd function argument include.root = FALSE]) and a mean value across the 1000 trees was calculated. Mean values were then rescaled, dividing by the total branch length sum across the tree including all 228 species and scales between zero (no species) and one (all species).

Functional diversity (technically, functional richness or trait space volume) was calculated using trait information (Table S4) and the volume of the multidimensional minimum convex hull (FRic) constructed using the dbFD function in the R package FD (Laliberté et al., 2014). The results were scaled relative to the volume of the hull containing all species in the entire dataset (across all regions and times), so functional diversity metrics vary between a theoretical minimum of zero (no species) and maximum of one (all species). The calculations used a five‐dimensional trait space constructed following principal coordinates analysis using Gower dissimilarity based on the trait values of all 228 species. Input variables were standardized and equal weighting assigned to each variable grouping by trait type (e.g., the seven diet category variables were assigned a 1/7 weight as diet was considered a single trait, Table S4). Square root transformation was used due to the resulting non‐Euclidean distance matrix (Supporting Information Methods and Table S4). Reducing dimensionality reduces the total amount of information available (Table S6), but the hull volume methods in the FD package require species richness to be greater than the number of functional space dimensions. The results were not found to be sensitive to the number of dimensions used (Table S6). The same axes were used to calculate the proportion of functional space occupied in 8000 BP that was still occupied in 2020 using convex hull intersections in the betapart package v. 1.5.4 (Baselga et al., 2021).

Thus, all three metrics of diversity potentially scale between zero (no species) and one (for a theoretical assemblage including all extant and extinct species), thus aiding in interpretation and comparisons of the values produced. The use of the three types of metrics together provides important insight into community changes; functional diversity may only reflect certain niche elements whereas phylogenetic diversity can potentially represent a wider range of characters as well as future potential (Owen et al., 2019).

For the purpose of the final analyses (potential for further re‐establishment), domestic breeds were treated as having the same traits and phylogenetic values as their extinct ancestors (e.g., Heck or longhorn cattle for auroch [*Bos primigenius*], Konik or Exmoor ponies for tarpan [*Equus ferus*]).

## 3 RESULTS

### 3.1 Continental scale

Considering all regions together (the “whole continent”), we estimate that 198 species of mammal were present 8000 years ago, which increased to 211 species in the year 2020, of a total of 228 species recorded over the entire period. Scaled taxonomic species richness thus increased from 0.87 to 0.93.

Seventeen species were extirpated from every region (i.e., from Europe): Twelve of these species are now globally extinct (Tables S5 and S7), while four still have extant populations in Asia and/or Africa (*Panthera leo*, *P. pardus*, *P. tigris* and *Saiga tatarica*; Crees, Turvey, et al., 2019; IUCN, 2021b). This leaves *Equus ferus* which is survived by the subspecies *Equus ferus przewalskii* (IUCN, 2021b) and domesticated descendants. The species that were recorded as becoming globally extinct were mostly restricted to islands (some may have had wider distributions in the Pleistocene) or those which were the focus of hunting and/or domestication. The only species that were distributed across the continental mainland 8000 years ago that are now globally extinct are *Bos primigenius* and *Equus hydruntinus* which both have surviving closely related or domesticated species (Table S5).

By contrast, 30 species were recorded as new introductions. These species ranged from intentional introductions, such as many deer species, to others whose introduction and spread were facilitated but likely unintentional (e.g., several rodent species).

This species turnover increased net phylogenetic diversity for the continent from 0.82 to 0.96 and functional diversity from 0.94 to 0.98. The 2020 species richness, phylogenetic diversity, and functional diversity values are, therefore, comparable to or slightly greater than those of 8000 years ago, at this continental scale.

### 3.2 Regional scale

**FIGURE 1:**
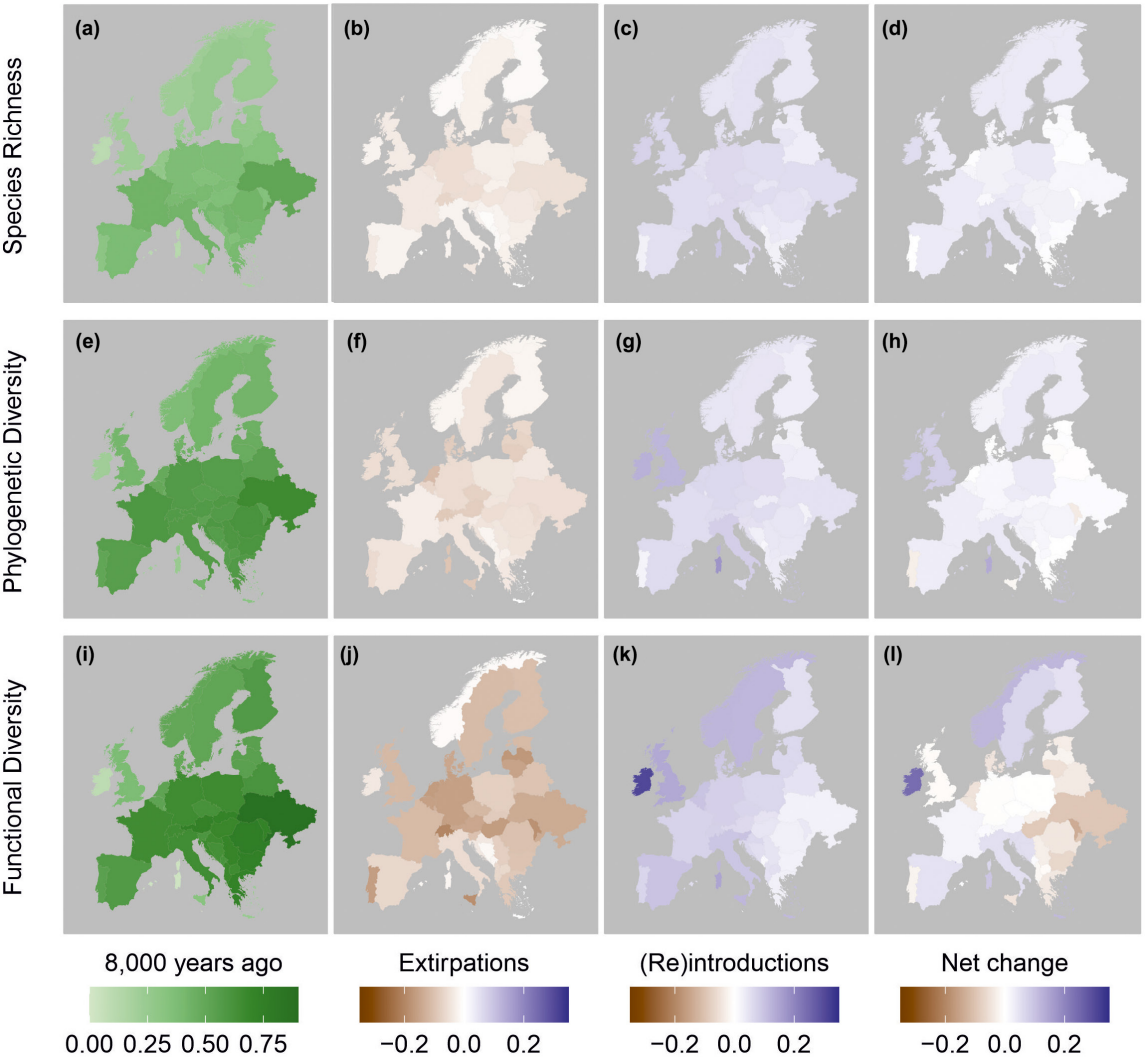
Map of species richness, phylogenetic diversity, and functional diversity for each region. Panels show estimated values 8000 years ago, change associated with extirpations (losses), change associated with introductions, reintroductions and other range expansions (gains), and overall change (net change). See the methods and Tables S2 and S3 for information on distribution change categories and how they were combined. Region outlines were obtained from gadm.org. Map lines delineate study areas and do not necessarily depict accepted national boundaries.

The number of species (richness) present increased over the 8000‐year period in 41 of the regions considered, decreased in one region, and showed no net change in three regions (Figures 1 and 3), representing a median scaled richness increase of 0.02 (range −0.004 to 0.07; a median increase of five species, range −1 to +16 species). Phylogenetic diversity increased in 33 regions and decreased in 12 with a median change of 0.02 and range of −0.04 to 0.14 (Figures 1 and 3). Functional diversity changes comprised both increases (20 regions) and decreases (25 regions), with a median change of −0.005 and a range from −0.14 to 0.24 (Figures 1 and 3). Thus, median functional diversity did not change, but the differences between regions are the greatest for this metric (some regions showing large function diversity increase, others decline). The functional spaces occupied by the species community 8000 years ago and in 2020 generally showed a high level of overlap, with the median proportion of the “older” community functional space still occupied being 0.9, the only major exceptions being some island regions (Table S8).

**FIGURE 2:**
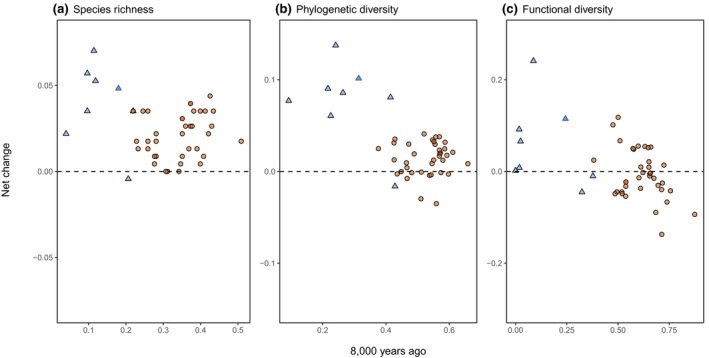
Species richness, phylogenetic diversity, and functional diversity estimates for each region 8000 years ago compared to net change between 8000 years ago and 2020. Islands and island groups are shown as light blue triangular points and continental regions as light brown circular points.

**FIGURE 3:**
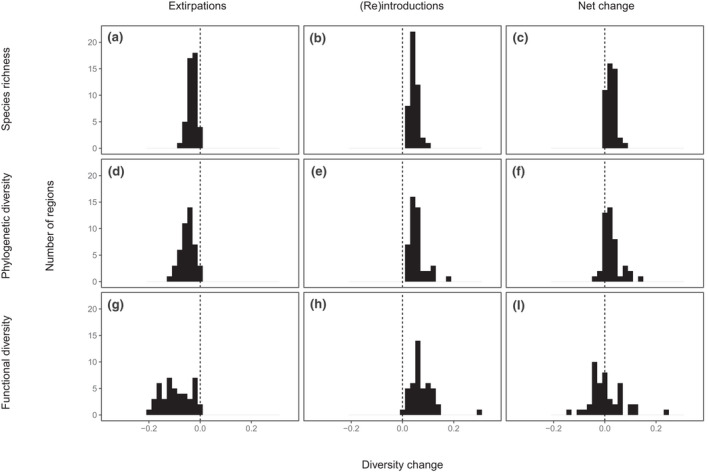
Histograms showing the distribution of species richness, phylogenetic diversity, and functional diversity losses due to extirpations, gains due to introductions, reintroductions, and other range expansions and net change estimates over the last 8000 years.

Regions with the lowest diversity 8000 years ago generally showed the greatest increases by 2020 (Figures 1 and 2) for all three metrics of diversity, with the relatively modest net changes explained by the competing effects of faunal losses and gains (Figures 1 and 3). The increase in species richness can be explained by the fact that more species were introduced than were extirpated. Phylogenetic diversity increased on most islands, as “missing” lineages were introduced, but showed smaller changes for continental regions (Figures 1 and 2). The relatively small changes in functional diversity are likely due to functional overlaps among species, including between some of the extirpated and introduced species.

Given the levels of environmental change, species introductions, and reintroductions since the mid‐20th century, we conducted a supplementary analysis of changes between 1945 and 2020 to identify “Anthropocene” trends. These show gains for most regions for all three diversity measures, primarily associated with mammal recoveries and introductions since the mid‐20th century (Figure S1).

When considering only large (>2 kg) non‐volant mammals, the distribution of regional net diversity change comparing 2020 and 8000 years ago was similar to when the whole community was considered (Figure S4; especially when considering phylogenetic [Figure S4b] and functional [Figure S4c] diversity). Small (<2 kg) non‐volant mammals showed increased diversity values in 2020 compared to 8000 years ago for nearly all regions (Figure S4). Species richness patterns indicate that this could be due to gains in small species with predominantly plant‐based diets (Figure S6). Caution must be taken here due to the quality of small mammal data in historic and zooarchaeological data (Crees, Collen, & Turvey, 2019).

### 3.3 Future re‐establishment

**FIGURE 4:**
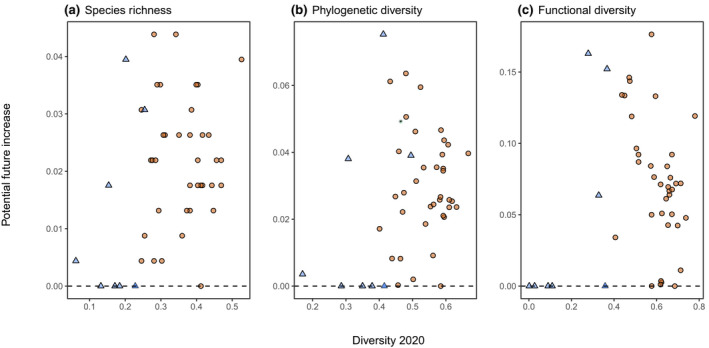
Future potential increases in species richness, phylogenetic diversity, and functional diversity for each region if all possible reintroductions take place compared with diversity values estimated for 2020 (dashed line = no increase). Islands and island groups are shown as light blue triangular points and continental regions as light brown circular points.

If all of the species that are still globally extant could recolonize (or be reintroduced to) the regions from which they have been extirpated, and if living relatives of extinct species were restored (including domesticated descendants that could be used to establish feral populations in regions where their wild ancestors existed), this would result in species richness, phylogenetic diversity, and functional diversity increases in 40 regions (five would remain unchanged; Figure 4). The unchanged regions are mostly islands for which historic losses were species‐level extinctions (i.e., there are no extant populations available to restore). The median change in species richness would be an increase of 0.02 (5 species) with a range of 0–0.04 (0–10 species). Phylogenetic diversity median change would be an increase of 0.03 (range 0–0.08). Functional diversity median change would be an increase of 0.07 with a range of 0–0.18. Thus, the greatest potential gains from re‐establishing species relate to functional diversity. In addition, more regions had the potential for the reintroduction of large (>2 kg) when compared to small (<2 kg) non‐volant mammals (Figure S5).

### 3.4 Relationships between metrics

All three metrics showed high correspondence (Figure S2). Phylogenetic and functional diversities were linearly related to one another (Figure S2, top‐middle panel), which is expected because phylogenetically distinct species are more likely to be functionally distinct. In contrast, species richness showed a slightly curvilinear relationship with the other two metrics, indicating that there is an increased likelihood of phylogenetic (e.g., congeners) and functional overlap among species at high‐species richness values (Figure S2, top‐left, top‐right). All three change value measures are also positively correlated with one another (Figure S2, bottom row).

## 4 DISCUSSION

The results presented here identify the importance of considering both losses and gains of biodiversity, and of doing so for different aspects of biodiversity. Our findings confirm the role of extirpations on species diversity and extend them for phylogenetic and functional diversity—the disappearance of species inevitably decreases diversity measured here at continental and within‐continent (regional) scales. However, the familiar narrative of species losses is only a portion of the story: species colonizations, introductions, and recoveries generate additions to all three metrics of diversity. When these species gains are considered alongside extirpations, net diversity is increased and restored for all three metrics across Europe as a whole (treating the parts of Europe considered here as one entity). Moreover, overall mammalian species richness increased in most separate regions (countries, island groups) within the continent, and phylogenetic diversity increased in more than two‐thirds of those regions, while functional diversity increased in some regions and declined in others. Species, phylogenetic, and functional turnover have taken place during the Holocene, but this has not generally resulted in a net attrition of these measures of diversity for the European mammal fauna, at the scales studied here.

These results are akin to the observation that increased turnover in the species composition of local ecological communities over the last century has rarely resulted in net declines in species richness (Dornelas et al., 2014; Vellend et al., 2013). Likewise, regional‐scale analyses suggest that plant species richness has increased in many, and probably most, regions of the world (Ellis et al., 2012; Sax et al., 2002; Vellend et al., 2017). By considering the functional diversity consequences of species turnover (and the implied functional consequences of phylogenetic diversity), our results support the contention that introduced mammal species can restore functions lost with extinct species (Lundgren et al., 2020).

The results also highlighted geographic variation in patterns of faunal change. Although such results should be interpreted with caution due to heterogeneous research efforts and data availability (Crees, Collen, & Turvey, 2019), they revealed a degree of geographic variation in the patterns of change on the European continental mainland. Net functional diversity tends to increase in western Europe, showing no net change in central areas, but declines in the east (Figure 1; species richness and phylogenetic changes were somewhat more evenly distributed across the continental mainland but still tended to show the greatest gains in the west). Several factors may contribute to this, including the climate (the Atlantic fringe of Europe has relatively mild winters), colonial and trading histories (seafaring nations in western Europe imported many species; Lenzner et al., 2018), the extent and age of reintroduction programs (Deinet et al., 2013), and dispersal of introduced species through Europe after their initial establishment (Tedeschi et al., 2022).

The strongest geographic pattern, however, was that islands showed the greatest faunal and functional turnover and exhibited the greatest net increases in all three diversity metrics. This may partly be associated with the increased susceptibility of native island species to extirpation or extinction, and greater potential for invasion, but it was difficult to identify from our data whether this was an island effect per se or simply a consequence of their functionally depauperate initial condition (Figure 2). Most of the island mammal communities considered in this study were dominated by insectivores 8000 years ago, but now include a wider range of carnivores (predators of vertebrates) and herbivores, moving community diet composition closer to that observed for continental regions (Figure S3). Most small and relatively oceanic islands also saw an increase in the average species body mass (Figure S3), but this was not the case for larger, land‐bridge islands that historically shared meso‐ and megafaunal species with the continent (e.g., Sicily). Species, phylogenetic, and functional turnover on islands therefore presents a complex picture. Species and potentially functional diversity may increase for some taxa on islands (Sax et al., 2002), but functional diversity can be lost for a subset of taxonomic groups (e.g., as for island‐adapted endemic birds; Sayol et al., 2021). Mammalian island faunas are known to have been greatly altered by humans (Longman et al., 2018). Such changes have had major impacts on the vegetation of island and prey communities (Simberloff, 1995). Hence, the increasing “continentality” of island biotas can simultaneously increase within‐island diversity and decrease global gamma diversity. Such changes are also possible in continental regions, but the effect sizes are small (Staude et al., 2022).

It is important to emphasize that our results relate to the attributes of regional faunas rather than direct measurements of community composition at a site level, for which there are too few multispecies archaeological deposits to make realistic comparisons with the present day. Population sizes and distributions of many species have changed (Ceballos & Ehrlich, 2002; Crees et al., 2016; Temple & Terry, 2007), including both steep declines and major expansions, and hence community compositions and turnover are likely to be considerably more variable at a local scale. Nonetheless, large mammals can sometimes recolonize large areas (Chapron et al., 2014; Milanesi et al., 2017), and some species (e.g., keystone species) have large effects even in small numbers, and so the presence of species in a region still has ecological relevance.

The current species present in any region represents the existing pool from which local communities are drawn most readily, enabling them to respond to and potentially form novel communities as a consequence of ongoing environmental changes. Additional species may recolonize from other regions (Chapron et al., 2014; Milanesi et al., 2017). The reintroduction of additional species and the establishment of feral populations of “primitive” breeds of “extinct” domesticated species provide opportunities for conservation reintroduction and rewilding programs to further increase all three metrics of diversity. This potential also appears to be higher for larger species concurring with the focus of many rewilding initiatives (Figure S5). With few known global extinctions of European mammal species in the last 8000 years (the 12 globally extinct species were endemic to particular island groups except for *Bos primigenius* whose widespread descendants are domestic cattle and *Equus hydruntinus* which is closely related to or even a subspecies of the extant *Equus hemionus* Bennett et al., 2017), a high proportion of the “original” functional space still exists somewhere in Europe or beyond (Tables S5 and S7). In principle, it would be possible to increase species, phylogenetic, and particularly functional diversities in nearly all continental regions and land‐bridge islands (Figure 4), and to achieve levels that are as high or higher than 8000 years ago—wherever space and other priorities permit. The species and communities will not be identical to those in the past—nor would we expect them to be under current climatic and other anthropogenic conditions—but there is considerable potential for the development of diverse mammal communities.

Diversity metrics alone, even when considering phylogenetic and functional aspects, are still unlikely to capture all the complexity of environmental management and conservation decision‐making. As mentioned above, the presence of a particular species in a region does not necessarily mean that it will fulfil a number of possible specific goals, such as ensuring population connectivity or maintaining disturbance regimes, especially if individuals are few, restricted to fenced reserves or only survive in remote locations with lower human population density (e.g., mountains). Reintroduction of modest numbers of ecosystem engineers such as beavers (*Castor fiber*), mega‐herbivores, or top predators may have disproportionate effects. The gain of some species may also be undesirable if their presence is detrimental to other species, especially those of cultural or conservation importance. Full accounting of biodiversity from all sources (losses and gains) is, however, extremely important to the understanding of species extinction risk in dynamic systems as well as in the discussion of conservation values (Wallach et al., 2020). The analysis presented here demonstrates the potential to increase diversity metrics relative to current and past baselines. It also highlights the need for discussion of the social values (high, low, or indifferent) that might be attributed to projects which aim to increase species, phylogenetic, or functional diversity and their consequences.

The results of this study suggest that the taxonomic, phylogenetic, and functional diversity losses of mammals over the last 8000 years have largely been replaced by immigrant species or recovered with reintroductions, with the largest net changes taking place on islands. These regional‐scale analyses and the further potential to introduce extirpated species and domesticated descendants of “extinct” species highlight the capacity to maintain and restore functionally and phylogenetically diverse mammal biotas. These biotas may even exceed diversity levels seen in the mid to late Holocene, and in many parts of Europe they already do.

## AUTHOR CONTRIBUTIONS

J.H.H. conducted the species occurrence data compilation and diversity analyses; K.E.D. researched and modified the phylogenetic trees. All authors contributed to research ideas and design and to the writing of the final manuscript.

## CONFLICT OF INTEREST

The authors declare no conflict of interests.

## Supporting information


Appendix S1

## Data Availability

All data are available from the sources cited. Species range map data and API access for the IUCN Red List need to be requested from the IUCN Red List of Threatened Species directly (https://www.iucnredlist.org/resources/spatial‐data‐download; https://apiv3.iucnredlist.org/about). Analysis code and calculated diversity metrics are available at https://doi.org/10.5281/zenodo.6673884.
