# Supplementary material for: Lost, gained, and regained functional and phylogenetic diversity of European mammals since 8000 years ago
Source: Glob Chang Biol. 2022 Jul 7;28(17):5283–93. doi: 10.1111/gcb.16316 (PMC9540530; doi:10.1111/gcb.16316)
Supplement: Supplementary file 1 — Appendix S1 [file GCB-28-5283-s001.docx]

**Lost, gained and regained functional and phylogenetic diversity of European mammals since 8,000 years ago**

Jack H Hatfield, Katie E Davis, Chris D Thomas

**Supporting Information**

**Methods**

*Regions*

Regions were downloaded in RDS format from GADM v3.6 (<https://gadm.org/>). Splits and combinations based on geography and historic groupings present in the species data are detailed in Table S1. The modified region polygons were then intersected with the species range maps (IUCN, 2021a) using st_intersection() in the R package sf (Pebesma, 2018), retaining the WGS 84 reference system due to the spatial extent.

**Table S1** Regions used detailing the GADM polygons and the abbreviation used.

| **Region** | **GADM units** | Abbreviation |
| --- | --- | --- |
| Aegean and Crete | Aegean and Crete (Greece) | AECR |
| Albania | Albania | ALB |
| Andorra | Andorra | AND |
| Austria | Austria | AUT |
| Balearic Islands | Islas Baleares (Spain) | BALE |
| Belgium | Belgium | BEL |
| Bulgaria | Bulgaria | BGR |
| Bosnia and Herzegovina | Bosnia and Herzegovina | BIH |
| Belarus | Belarus | BLR |
| Switzerland | Switzerland | CHE |
| Corsica | Corse (France) | CORS |
| Czechia | Czech Republic | CZE |
| Germany | Germany | DEU |
| Denmark | Denmark | DNK |
| Spain | Spain without Islas Canarias, Ceuta y Melilla and Islas Baleares | ESP |
| Estonia | Estonia | EST |
| Finland | Finland | FIN |
| France | France without Corse | FRA |
| Great Britain | United Kingdom without Northern Ireland | GBR |
| Greece | Greece without Aegean and Crete | GRC |
| Croatia | Croatia | HRV |
| Hungary | Hungary | HUN |
| Island of Ireland | Ireland (Ireland) and Northern Ireland (United Kingdom) | IoI |
| Italy | Italy without Sardegna and Sicily | ITA |
| Liechtenstein | Liechtenstein | LIE |
| Lithuania | Lithuania | LTU |
| Luxembourg | Luxembourg | LUX |
| Latvia | Latvia | LVA |
| Moldova | Moldova | MDA |
| North Macedonia | Macedonia | MKD |
| Malta | Malta | MLT |
| Montenegro | Montenegro | MNE |
| Netherlands | Netherlands | NLD |
| Norway | Norway | NOR |
| Poland | Poland | POL |
| Portugal | Portugal without Azores and Madeira | PRT |
| Romania | Romania | ROU |
| Sardinia | Sardegna (Italy) | SARD |
| Sicily | Sicily (Italy) | SICI |
| San Marino | San Marino | SMR |
| Serbia and Kosovo | Serbia and Kosovo | SRB_XKO |
| Slovakia | Slovakia | SVK |
| Slovenia | Slovenia | SVN |
| Ukraine | Ukraine | UKR |

*Older and recent time periods*

For the analyses presented in Figure S1 we looked at an additional 1945 divide with all changes happening in 1945 or later classed as recent and those before as older. This date was chosen as it is at the beginning of the post-war Great Acceleration with implications for energy and resource use, international trade, transit and globalisation, and its link to ‘the Anthropocene’ (McNeill and Engelke, 2014; Monastersky, 2015; Voosen, 2016; Ellis, 2018).

Dates for establishment/ extirpation of each species in each region were taken initially from the source providing the information on the range change (e.g., DAISIE) but where information was unavailable or could not be resolved (i.e., because only a date range was available), additional literature searches were conducted (Additional Sources). Date ranges that could not be refined and overlapped the 1945 boundary were assigned a time period based on the midpoint (e.g., for the establishment date of an introduced species). Where direct period evidence was lacking, we were able to use knowledge of the species distributional history and patterns for neighbouring regions to assign a time period. For example, the introduction of *Mus musculus* is known to be well within the older period (Cucchi *et al*., 2005; Genovesi *et al*., 2009). Other examples include the extirpation of *Castor fiber* in Montenegro where dates were not available, so extirpation was assigned to the older period based on Serbia and other surrounding regions (Halley *et al*., 2021). Diversity metrics for the recent and older time periods are provided in the online repository, revealing the strength of net increases in the post-1945 period.

*Globally extinct or largely domesticated species and reintroductions*

*Equus ferus*, *E. hydruntinus* and *Bos primigenius* were considered as reintroduction candidates. Feral horses could be used as a substitute for *E. ferus* alongside *Equus ferus przewalskii*, whose origins are disputed (Naundrup and Svenning, 2015; IUCN, 2021b; Smith *et al.,* 2021) and domestic cattle (*B. taurus*) a substitute for *B. primigenius*. *E. hydruntinus* is closely related (possibly even conspecific (Bennett et al., 2017)) to the extant *E. hemionus* so this was assumed to be a suitable substitute.

**Table S2** The categorical groups into which species were classified based on changes in their distribution over the last 8,000 years. All categories are mutually exclusive except Potential Reintroduction which can apply in addition to any of the extirpation categories.

| Category | Definition |
| --- | --- |
| Native | Species that have been present in a region from at least 8,000 years ago and that evidence suggests have been present throughout. |
| Older Extirpation | Species that were present in a region 8,000 years ago but then became extirpated before 1945. These species are no longer present. In some cases this may also represent their global extinction. |
| Recent Extirpation | Species that were present in a region 8,000 years ago but then became extirpated between 1945 and 2020. These species are no longer present. In some cases this may also represent their global extinction. |
| Unknown Extirpation | Species that were present in a region 8,000 years ago but have since become extirpated before 2020 (but status in 1945 is unknown). These species are no longer present. In some cases this may also represent their global extinction. |
| Older Reintroduction | Species that were present in a region 8,000 years ago, then became extirpated but were afterwards reintroduced or re-colonised before 1945. These species are currently present. |
| Recent Reintroduction | Species that were present in a region 8,000 years ago, then became extirpated but were afterwards reintroduced or re-colonised between 1945 and 2020. These species are currently present. |
| Older Introduction | Species that were not present in a region 8,000 years ago but were introduced before 1945. These species are currently present. |
| Recent Introduction | Species that were not present in a region 8,000 years ago but were introduced between 1945 and 2020. These species are currently present. |
| Recent Range Expansion | Species that evidence suggests were not present in a region 8,000 years ago but expanded into the region between 1945 and 2020. These species are currently present and differ from introductions as these are classed as movements thought not to be directly human aided. |
| Potential Reintroduction | Species that were present in a region 8,000 years ago but then became extirpated. These species however are still globally extant or have suitable relatives (Methods) meaning that they could theoretically be reintroduced in future. |

* Any species that may have colonised or been introduced after 8,000 years ago, but that were extirpated again or failed to successfully establish themselves before 2020 were excluded.

**Table S3** Tables showing how the different categories of distribution change (Table S2) are combined to form the assemblages for each region at each time point and also how losses and gains are calculated.

| Assemblage | Native | Older Extirpation | Recent Extirpation | Unknown Extirpation | Older Reintroduction | Recent Reintroduction | Older Introduction | Recent Introduction | Recent Range Expansion | Potential Reintroduction |
| --- | --- | --- | --- | --- | --- | --- | --- | --- | --- | --- |
| Time Period Start | 1 | 1 | 1 | 1 | 1 | 1 | 0 | 0 | 0 | 0 |
| Only Loses | 1 | 0 | 0 | 0 | 0 | 0 | 0 | 0 | 0 | 0 |
| Only Gains | 1 | 1 | 1 | 1 | 1 | 1 | 1 | 1 | 1 | 0 |
| 1945 | 1 | 0 | 1 | 0 | 1 | 0 | 1 | 0 | 0 | 0 |
| Recent Loses Only | 1 | 0 | 0 | 0 | 1 | 0 | 1 | 0 | 0 | 0 |
| Recent Gains Only | 1 | 0 | 1 | 0 | 1 | 1 | 1 | 1 | 1 | 0 |
| Time Period End | 1 | 0 | 0 | 0 | 1 | 1 | 1 | 1 | 1 | 0 |
| Future Potential | 1 | 0 | 0 | 0 | 1 | 1 | 1 | 1 | 1 | 1 |

*Taxonomy*

Taxonomy was aligned to the mammal phylogeny (Upham et al., 2019) obtained via vertlife.org with mismatches resolved using taxonomic information available from the IUCN (IUCN, 2021b), PHYLACINE (Faurby and Svenning, 2015; Faurby et al., 2018; 2020), itis.gov (Integrated Taxonomic Information System) and additional studies where required (Benda et al., 2004; Bover and Alcover, 2008; Juste et al., 2013; Cserkész et al., 2016).

*Species Traits*

Trait data was collected from the EltonTraits 1.0 dataset (Wilman et al., 2014; Smith et al., 2003) and the IUCN Red List of Threatened Species (IUCN, 2021b). Body mass values were log transformed, the diet percentages composed of vertebrates collapsed to a single variable and categorical foraging strata converted to four binary variables. Compilation of habitat types used the IUCN Red List API and the R package rredlist (Chamberlain, 2020) to obtain the information from the IUCN Red List (IUCN, 2021b). The data was used to produce binary variables with the following adjustments. The semi-open habitats of shrubland and savannah were combined, and a coastal category was formed from the combination of marine intertidal and marine coastal/supratidal. Marine neritic, marine oceanic and marine deep ocean floor were excluded as they were not relevant to the terrestrial focus. The introduced vegetation, other and unknown categories were also excluded.

For globally extinct and largely domesticated species body mass values were taken from PHYLACINE (Faurby and Svenning, 2016; Faurby et al., 2018; 2020; Smith et al., 2003)), and from the wider literature or based on genus and family averages when a suitable estimate was lacking (Table S5).

Where a trait was actually composed of multiple variables (e.g., diet being represented as a percentage across seven resource types), weighting was used to reduce the chance of a particular variable group contributing disproportionately to functional space (Table S4).

**Table S4** Species trait variables used in functional diversity analyses.

| Traits | Data Type | Format | Weight | Main Source |
| --- | --- | --- | --- | --- |
| Body mass | Continuous | One variable. Natural log of body mass | 1 | Elton Traits; Smith et al., 2003 |
| Activity Period | Asymmetric binary | Three variables. Binary for diurnal, nocturnal and crepuscular. | 1/3 | Elton Traits |
| Foraging Strata | Asymmetric binary | Four variables. Converted to binary for ground, scansorial, arboreal and aerial. | 1/4 | Elton Traits |
| Diet | Continuous | Seven variables. Percentage diet composed of vertebrates, invertebrates, scavenging, fruit, seeds, nectar and of other plant material. | 1/7 | Elton Traits |
| Habitat Types | Asymmetric binary | Ten variables. Binary for forest, scrubland or savannah, grassland, wetlands (inland), rocky areas, caves and subterranean, desert, coastal, artificial terrestrial and artificial aquatic. | 1/10 | IUCN |

**Table S5** Sources of trait values used for globally extinct or domesticated species.

| Species | Historic distribution | Trait sources |
| --- | --- | --- |
| *Mus minotaurus* | Crete (Turvey, 2009) | Body mass based on the most recent time period estimate reported in van der Geer et al. (2013). Other traits based on genus average. |
| *Equus ferus* | Widespread in continental Europe (Crees *et al.,* 2019) | Body mass from PHYLACINE. Based on domesticated *Equus ferus caballus* (*Equus caballus*) in Elton Traits. Habitat types based on *Equus* average. Although the subspecies *Equus ferus przewalskii* is extant it has a comparatively small biogeographic range and its origins are unclear (IUCN, 2021b; Smith *et al*., 2021). |
| *Equus hydruntinus* | Widespread in continental Europe (Crees *et al.,* 2014) | Body mass from PHYLACINE. Other traits based on close relative *Equus hemionus* (Bennett et al. 2017). |
| *Myotragus balearicus* | Balearic Islands (Turvey, 2009) | Body mass from PHYLACINE. Other traits based on *Budorcas* taxicolor (Bover et al. 2019; Alcover & Bover, 2000). |
| *Asoriculus hidalgo* | Balearic Islands (Turvey, 2009) | Body mass from PHYLACINE. Listed in Elton Traits as *Nesiotites hidalgo*. Habitats based on *Soriculus nigrescens* (Bover & Alcover, 2008; Bover et al., 2018). |
| *Eliomys morpheus* | Balearic Islands (Turvey, 2009) | Body mass from PHYLACINE. Other traits based on genus average (Traveset et al., 2009; Bover et al., 2020). |
| *Prolagus sardus* | Corsica and Sardinia (Turvey, 2009) | Body mass from PHYLACINE. Other traits from the average of the Ochotona. |
| *Praemegaceros cazioti* | Corsica and Sardinia (Turvey, 2009) | Body mass from PHYLACINE. Other traits based on Cervidae average. |
| *Asoriculus corsicanus* | Corsica (Turvey, 2009) | Assigned the same values as *Asoriculus similis* |
| *Tyrrhenicola henseli* | Corsica and Sardinia (Turvey, 2009) | Body mass from PHYLACINE (*Microtus henseli*). Other traits based on average for *Microtus*. |
| *Asoriculus similis* | Sardinia (Turvey, 2009) | Body mass from PHYLACINE. Listed in Elton Traits as *Nesiotites similis*.  Habitats based on *Soriculus nigrescens* (Bover & Alcover, 2008; Bover et al., 2018). |
| *Rhagamys orthodon* | Corsica and Sardinia (Turvey, 2009) | Body mass from PHYLACINE. Listed in Elton Traits. Other traits based on *Apodemus* genus average following PHYLACINE phylogeny. |
| *Bos primigenius* | Widespread in Europe (Crees *et al.,* 2016) | Body mass from PHYLACINE. Based on *Bos taurus* in Elton Traits and listed by IUCN. |

*Functional Space Dimensions*

By reducing the multidimensional space created based on the functional traits to a fixed number of axes, the ability to fully represent the full space is also reduced (Table S6). To understand the influence of our choice to use a five-dimensional functional space on the results we first conducted a preliminary investigation using the mean squared deviation method and scripts provided in Maire *et al.* (2015) which showed only minor improvement with more dimensions. We then compared the functional diversity values obtained using additional dimensions to those we obtained using five. Pearson correlation coefficients between functional diversity calculated using five dimensions and using six and seven dimensions were 0.99 and 0.95 respectively.

**Table S6** Quality of reduced-space used for calculation of functional diversity compared when using different numbers of dimensions.

| Number of dimensions | Quality of reduced-space representation |
| --- | --- |
| 2 | 0.36 |
| 3 | 0.44 |
| 4 | 0.51 |
| 5 | 0.56 |
| 6 | 0.60 |
| 7 | 0.64 |

*Phylogeny Diversity*

A set of 1,000 node-dated trees, obtained from the distribution of trees published by Upham et al. (2019), were downloaded from vertlife.org. All globally extant taxa were matched to the appropriate tip labels. For the 12 globally extinct species, three (*Bos primigenius, Myotragus balearicus* and *Prolagus sardus*) were already in the phylogeny. The remaining nine were added to each of the 1,000 phylogenies via the following methodology. Position in the tree was determined by a thorough literature search for information on phylogenetic position and taxonomy for each taxon. Where possible, taxa were allocated a position according to previously published phylogenetic analyses, otherwise positions were assigned using taxonomy. Origination dates, to provide realistic branch lengths, were also obtained from the literature or from the Paleobiology Database (https://paleobiodb.org). To account for uncertainty in both topology and origination dates each taxon was added to each tree individually, randomly resolved (if resulting in a polytomy) then the branch length subtending that taxon was chosen from a uniform distribution between the First Appearance Datum (FAD and the Last Appearance Datum (LAD). This resulted in a set of 1,000 trees now containing the extinct taxa, each with differing topologies and dates. The extinct horse *Equus hydruntinus* was treated differently as there was only one other member of *Equus* in the phylogenies and no reliable fossil age data could be found. For this taxon, origination dates were obtained from the PHYLACINE phylogeny (Faurby and Svenning, 2015b; Faurby et al., 2018; 2020), these were then treated as a distribution from which a random date was allocated for each of the 1,000 phylogenies. All dates, except those obtained from PHYLACINE (Faurby and Svenning, 2015; Faurby et al., 2018; 2020), were checked against the most recent (March 2020) version of the ICS International Chronostratigraphy (Cohen et al., 2013). Finally, the tips of all 1,000 trees were pruned back to 8,000 years ago to provide an estimation of how the European mammal phylogeny would have appeared at this time and provide consistency in phylogenetic diversity measures.

PD was calculated using the mean value across 1,000 trees summing branch lengths (excluding the root, although root inclusion was not found to affect the results) using the pd function in R package picante (Kembel *et al*., 2010).

**Table S7** Information regarding the placement of species missing from the original phylogeny with the First (FAD) and Last Appearance Datum (LAD) values used and a description of species positions.

| **Extinct species and synonyms** | **Position** | **Age** | **FAD** | **LAD** | **Sources** |
| --- | --- | --- | --- | --- | --- |
| *Asoriculus corsicanus* (*Nesiotites corsicanus*) | Placed with other *Asoriculus* | Late Pliocene – Early Pleistocene | 3.6 | 0.774 | Bover *et al.,* 2018; Moncunill-Sole *et al*., 2016. |
| *Asoriculus hidalgo* (*Nesiotites hidalgo*) | Sister to *Neomys* | Middle Pleistocene – Holocene | 0.774 | 0 | Bover *et al.,* 2018; Moncunill-Sole *et al*., 2016. |
| *Asoriculus similis* (*Nesiotites similis*) | Placed with other *Asoriculus* | Early Pleistocene – Holocene | 2.58 | 0 | Bover *et al.,* 2018; Moncunill-Sole *et al*., 2016. |
| *Eliomys morpheus* (*Hypnomys morpheus*) | Sister to *Eliomys quercinus* | Late Pleistocene | 0.129 | 0.0117 | Bover *et al.,* 2019; PBDB; van den Hoek Ostende *et al.,* 2017; PHYLACINE |
| *Equus hydruntinus* (*Equus hemionus hydruntinus*) | Sister to *Equus ferus* |  | 5 | 0.67862 | PHYLACINE |
| *Mus minotaurus* | Not been placed in a phylogeny so placed at base of extant *Mus* as a conservative estimate | Lower Pleistocene | 2.58 | 1.8 | PBDB; van den Hoek Ostende *et al.,* 2017. |
| *Praemegaceros cazioti* | With *Axis axis, Dama dama, Cervus elaphus, Cervus nippon* – place in polytomy at base then randomly resolve x times | Late Pliocene – Late Pleistocene | 3.6 | 0.0117 | PHYLACINE; PBDB; Raia and Meiri, 2006. |
| *Rhagamys orthodon* | Within *Apodemus* – place in polytomy at base then randomly resolve x times | Middle Pleistocene – Holocene | 0.774 | 0 | PHYLACINE; PBDB; Salotti *et al*., 1997; Abbazzi *et al*., 2004. |
| *Tyrrhenicola henseli* (*Microtus henseli*) | Within *Microtus*, place at base in polytomy then randomly resolve x times | Middle Pleistocene – Holocene | 0.774 | 0 | PHYLACINE; PBDB; Salotti *et al*., 1997; Abbazzi *et al*., 2004. |

**Table S8** Functional space overlap between the start of the full time period (8,000 years ago) and the end of the time period (2020) for each region. These represent the intersection of the two functional hulls to identify the overlapping volume which is then provided as a proportion of the original functional volume (functional hull estimate for 8,000 years ago).

| Functional space overlaps | Region |
| --- | --- |
| 1.00 | AECR |
| 0.91 | ALB |
| 1.00 | AND |
| 0.94 | AUT |
| 0.38 | BALE |
| 0.75 | BEL |
| 0.88 | BGR |
| 1.00 | BIH |
| 0.87 | BLR |
| 0.90 | CHE |
| 0.46 | CORS |
| 0.90 | CZE |
| 0.89 | DEU |
| 0.75 | DNK |
| 0.94 | ESP |
| 0.84 | EST |
| 1.00 | FIN |
| 0.93 | FRA |
| 0.72 | GBR |
| 0.91 | GRC |
| 0.99 | HRV |
| 0.81 | HUN |
| 0.77 | IoI |
| 0.94 | ITA |
| 0.88 | LIE |
| 0.80 | LTU |
| 0.74 | LUX |
| 0.86 | LVA |
| 0.76 | MDA |
| 0.91 | MKD |
| 0.99 | MLT |
| 1.00 | MNE |
| 0.74 | NLD |
| 1.00 | NOR |
| 0.90 | POL |
| 0.86 | PRT |
| 0.91 | ROU |
| 0.30 | SARD |
| 0.63 | SICI |
| 0.91 | SMR |
| 0.89 | SRB_XKO |
| 0.90 | SVK |
| 0.94 | SVN |
| 0.93 | SWE |
| 0.87 | UKR |

*Species subgroups*

To understand the changes that have taken place in certain mammal groups and to provide more consistency in intra-group recording differences. we split species based on diet and body mass. Firstly, we separated out bats which as flying mammals may show different patterns. We split the remaining species based on body mass with species > 2kg being classed as large and those < 2kg small. This value was based on Crees *et al.* (2019) where 2kg is used due to limitations in the zooarchaeological record. We also split these size groups based on diet traits being used into those that predominantly eat animal-based sources (> 50% invertebrates, vertebrates, or scavenging) and those that predominantly eat plant-based sources (> 50% fruit, nectar, seeds, or other plant material). Where all three metrics were calculated (Figure S4 and S5) 6 regions (Aegean islands and Crete, Balearic Islands, Corsica, Island of Ireland, Malta, and Sardinia) were excluded due to one or more groups having too few species for metric calculation. For the same reason only species richness was calculated for bats and the diet splits. Functional spaces for the small and large mammal groups were constructed in the same way as for the full datasets and the quality of functional space was 0.57 and 0.41 respectively.

Table S9 – Median and range values of net change (8,000 years ago compared to 2020) across regions (39/45) for species richness, phylogenetic diversity and functional diversity. Calculated separately for small (< 2kg) and large (> 2kg) non-volant mammals.

| Subgroup | Metric | Regions median | Regions range |
| --- | --- | --- | --- |
| Small mammals | Species richness | 0.04 (+5 species) | 0.02 to 0.07 (+2 to +9 species) |
| Small mammals | Phylogenetic diversity | 0.07 | 0.01 to 0.11 |
| Small mammals | Functional diversity | 0.1 | - 0.01 to 0.18 |
| Large mammals | Species richness | 0 | - 0.07 to 0.07 (+4 to -4 species) |
| Large mammals | Phylogenetic diversity | - 0.009 | - 0.16 to 0.11 |
| Large mammals | Functional diversity | - 0.01 | - 0.36 to 0.16 |

Table S10- – Median and range values of potential future increases across regions (39/45) for species richness, phylogenetic diversity and functional diversity. Calculated separately for small (< 2kg) and large (> 2kg) non-volant mammals.

| Subgroup | Metric | Regions median | Regions range |
| --- | --- | --- | --- |
| Small mammals | Species richness | 0.008 (1 species) | 0 to 0.02 (0 to +3 species) |
| Small mammals | Phylogenetic diversity | 0.0005 | 0 to 0.04 |
| Small mammals | Functional diversity | 0 | 0 to 0.09 |
| Large mammals | Species richness | 0.07 (4 species) | 0 to 0.16 (0 to 9 species) |
| Large mammals | Phylogenetic diversity | 0.07 | 0 to 0.18 |
| Large mammals | Functional diversity | 0.07 | 0 to 0.36 |

*Software and resources*

Visual checking of shapefiles was conducted in QGIS (QGIS Development Team, 2021) with other analysis performed in R v.4.1.1  (R Core Team, 2021) with the additional packages ape v. 5.5 (Paradis and Schliep, 2019), betapart v. 1.5.4  (Baselga *et al*., 2021), clue v. 0.3-57 (Hornik, 2005; 2019), cluster v. 2.1.0 (Maechler *et al*., 2019), cowplot v. 1.1.1 (Wilke, 2020), dplyr v. 1.0.7 (Wickham *et al*., 2021), FD v. 1.0-12 (Laliberté and Legendre, 2010; Laliberté *et al*., 2014), geometry v. 0.4.5 (Habel *et al*., 2019), ggdist v. 3.1.1 (Kay, 2021), ggplot2 v. 3.3.5 (Wickham, 2016), gtools v. 3.8.2 (Warnes *et al*., 2020), picante v. 1.8.2 (Kembel *et al*., 2010), plyr v. 1.8.6 (Wickham, 2011), RColorBrewer v. 1.1-2 (Neuwirth, 2014), reshape2 v. 1.4.4 (Wickham, 2007), rredlist v. 0.7.0 (Chamberlain, 2020), scales v. 1.1.1 (Wickham and Seidel, 2020), sf v. 0.9-8 (Pebesma, 2018) and stringr v. 1.4.0 (Wickham, 2019).

**Supplementary Figures**


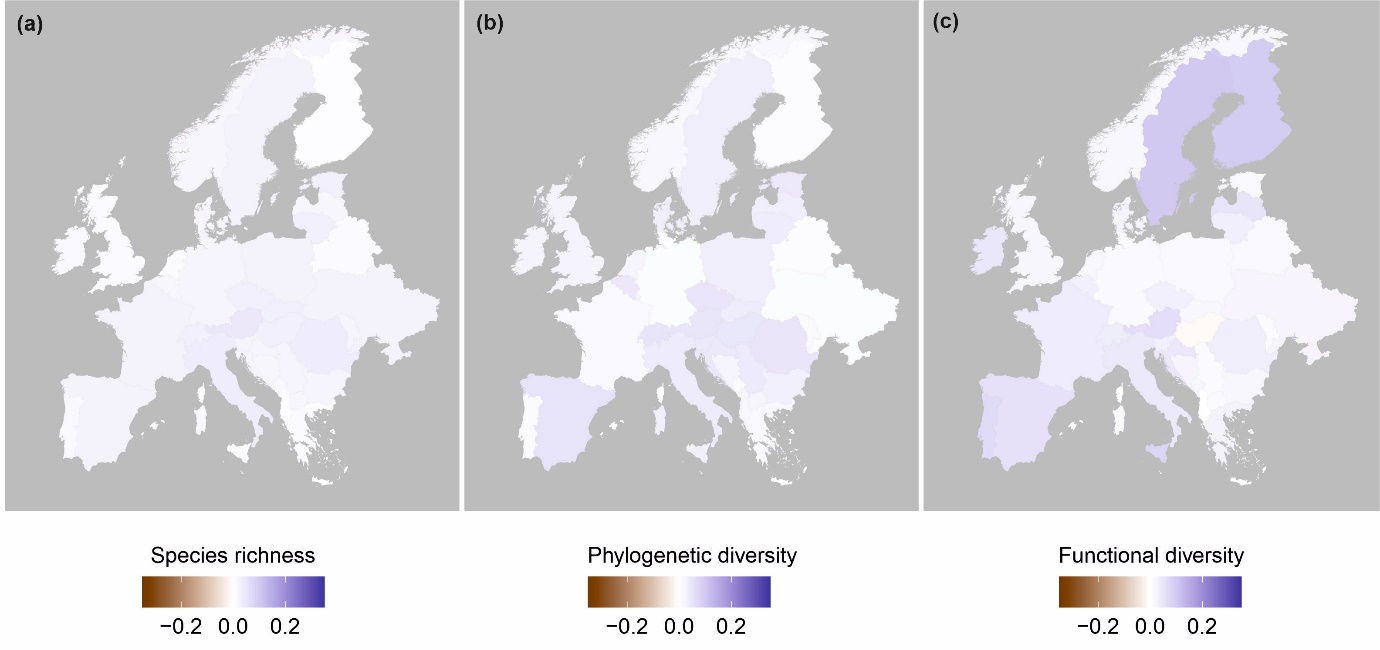


**Fig. S1** Changes in species richness, phylogenetic diversity and functional diversity between 1945 and 2020 for each region. Map lines delineate study areas and do not necessarily depict

accepted national boundaries.


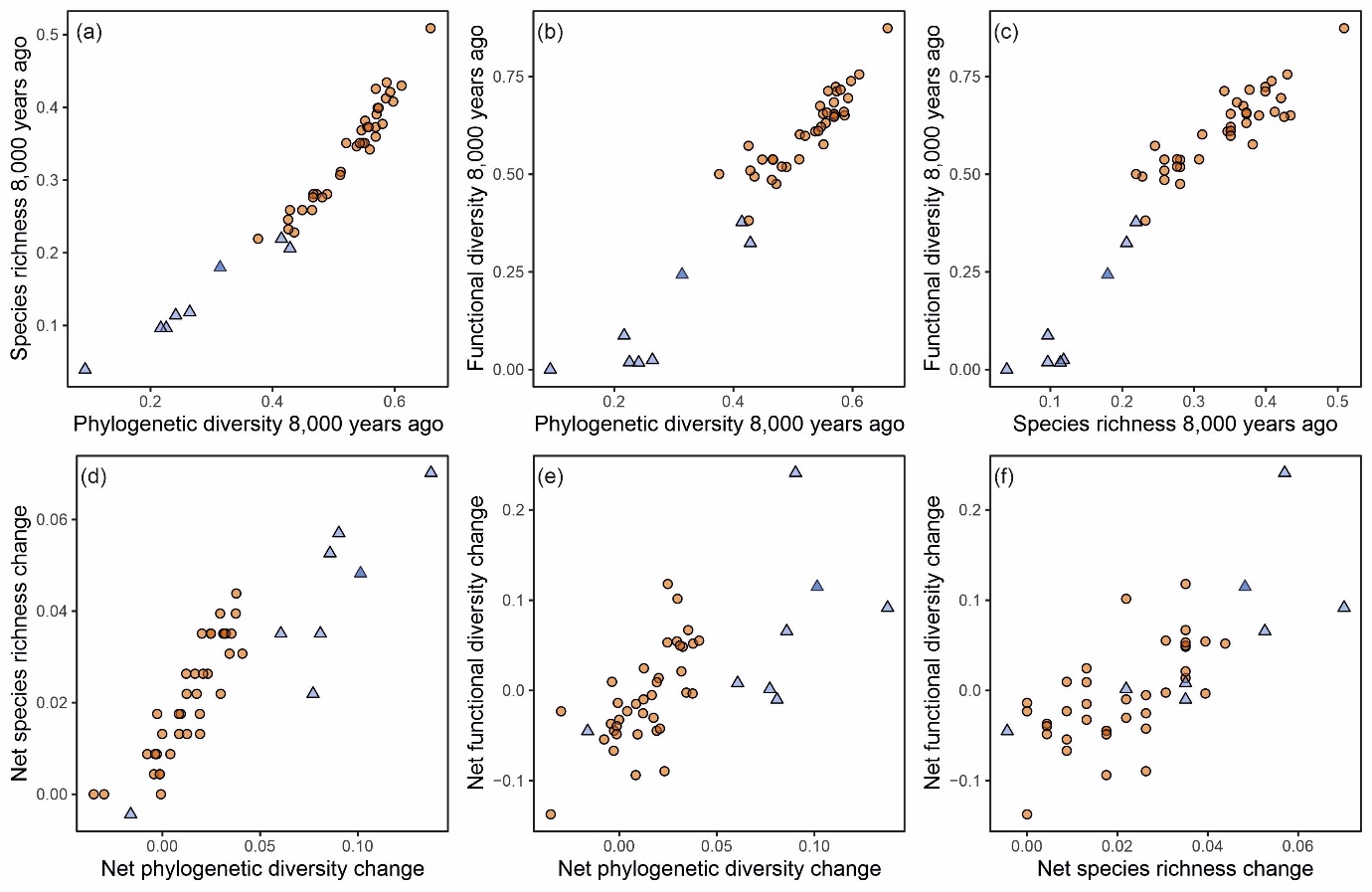


**Fig. S2** Comparisons of species richness, phylogenetic diversity and functional diversity for each region, considering values 8,000 years ago (upper) as well as net change over the time period (lower). Light brown circular points denote continental regions and light blue triangular points island regions.


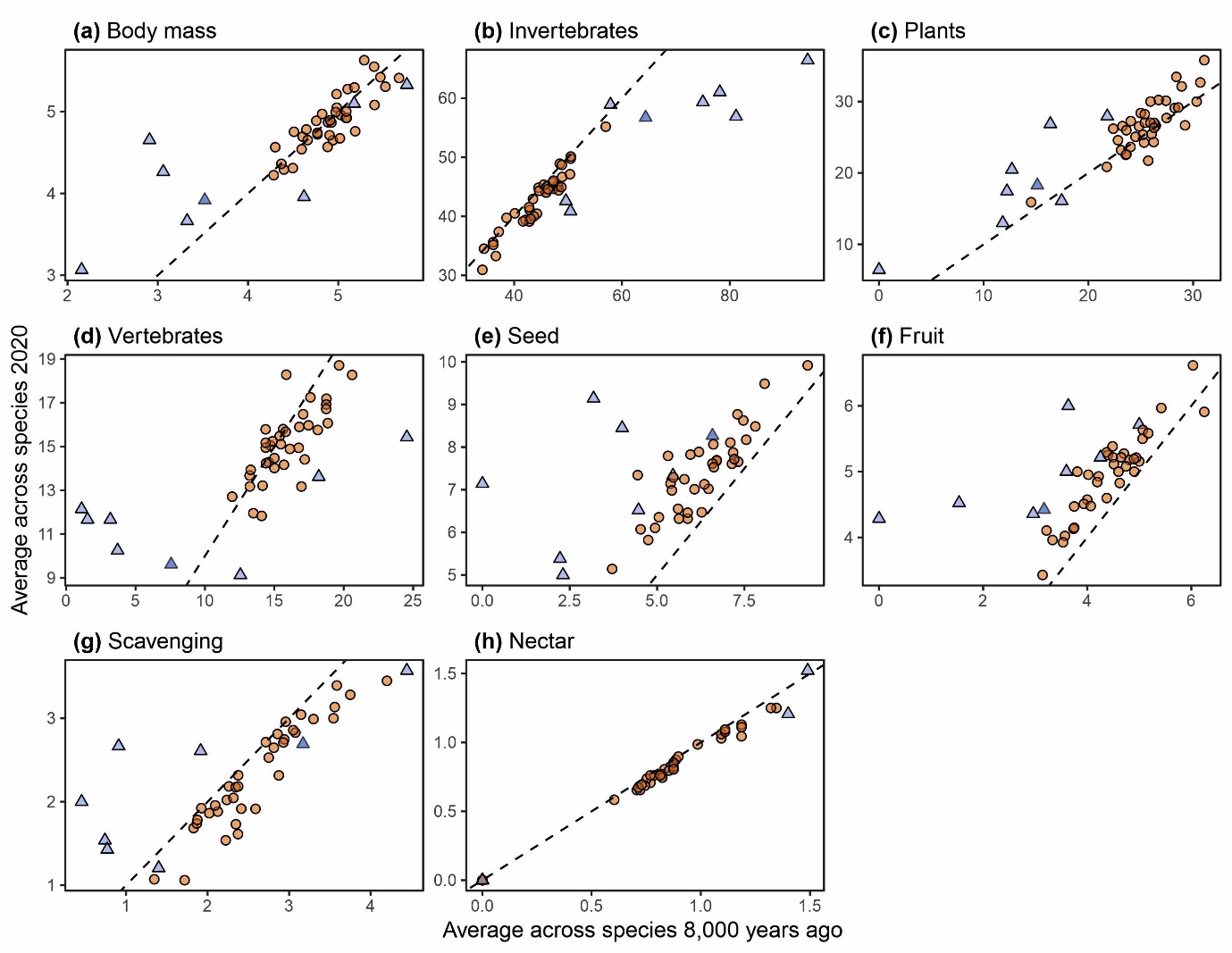


**Fig. S3** Community weighted means of body mass (a) and the seven diet categories (b – h). Body mass is shown as Ln(body mass in g) and diet categories are given percentage values with categories ordered by largest to smallest component. Each panel compares the values 8,000 years ago and in 2020. As occurrence data was used these correspond to the mean trait value across all species in the region's assemblage. Islands (n=8) are shown as light blue triangles and continental regions (n=37) as light brown circles. The dotted line represents no change (1:1).


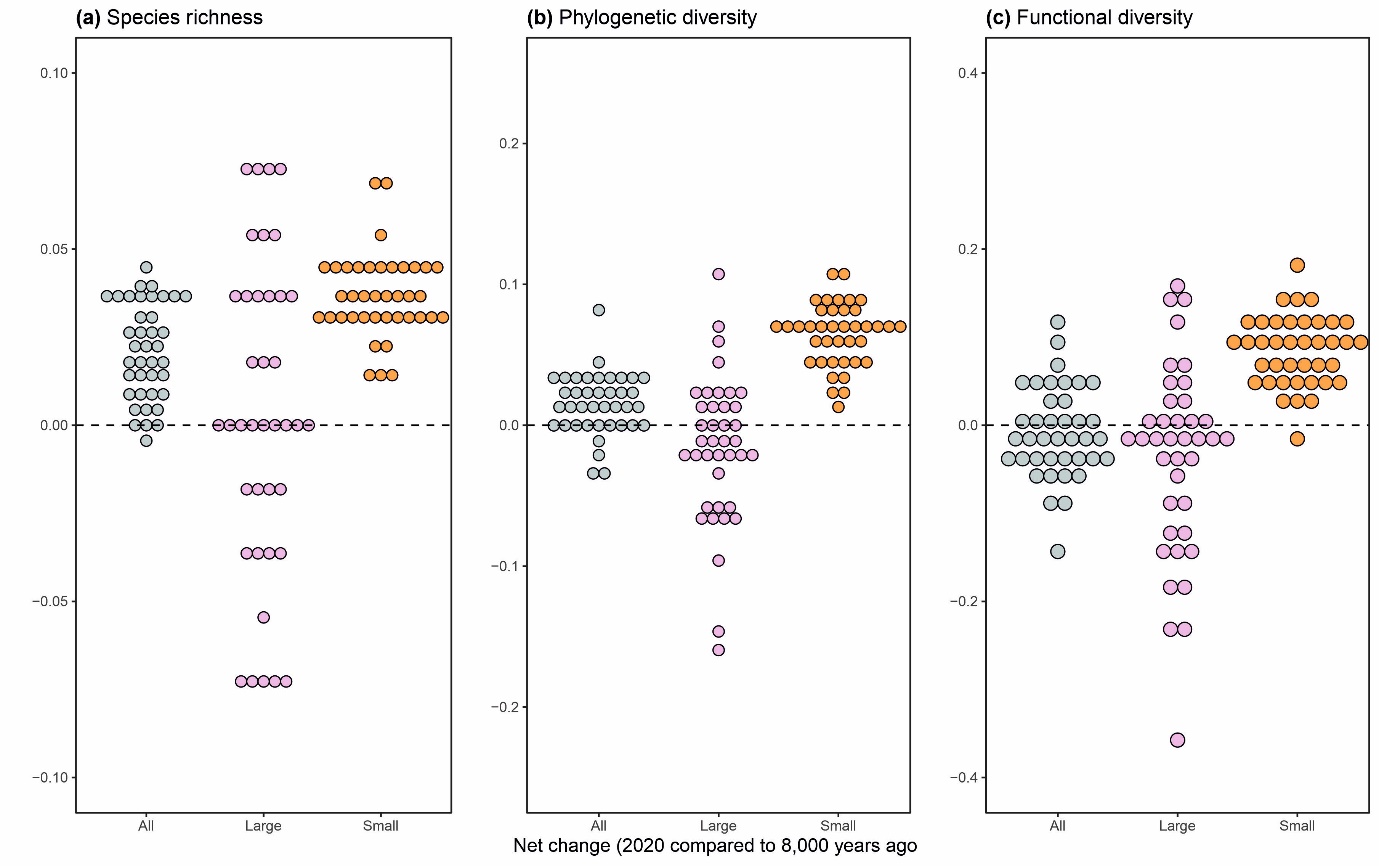


**Fig. S4** Net change in species richness (a), phylogenetic diversity (b) and functional diversity (c) comparing 8,000 years ago and 2020. Values considering all mammal species (including bats) are shown in grey, large mammal species (> 2kg and excluding bats) in plum and small mammal species (< 2kg and excluding bats) in orange. Values are scaled within group by the maximum obtainable values (e.g., for small mammals excluding bats, in proportion to the total diversity value for an assemblage containing all small mammals excluding bats). The dashed line represents zero change. Each dot represents a single region. The Aegean Islands and Crete, the Balearic Islands, Corsica, the island of Ireland, Malta and Sardinia were excluded here as they contained too few species in one or more groups for metric calculation.


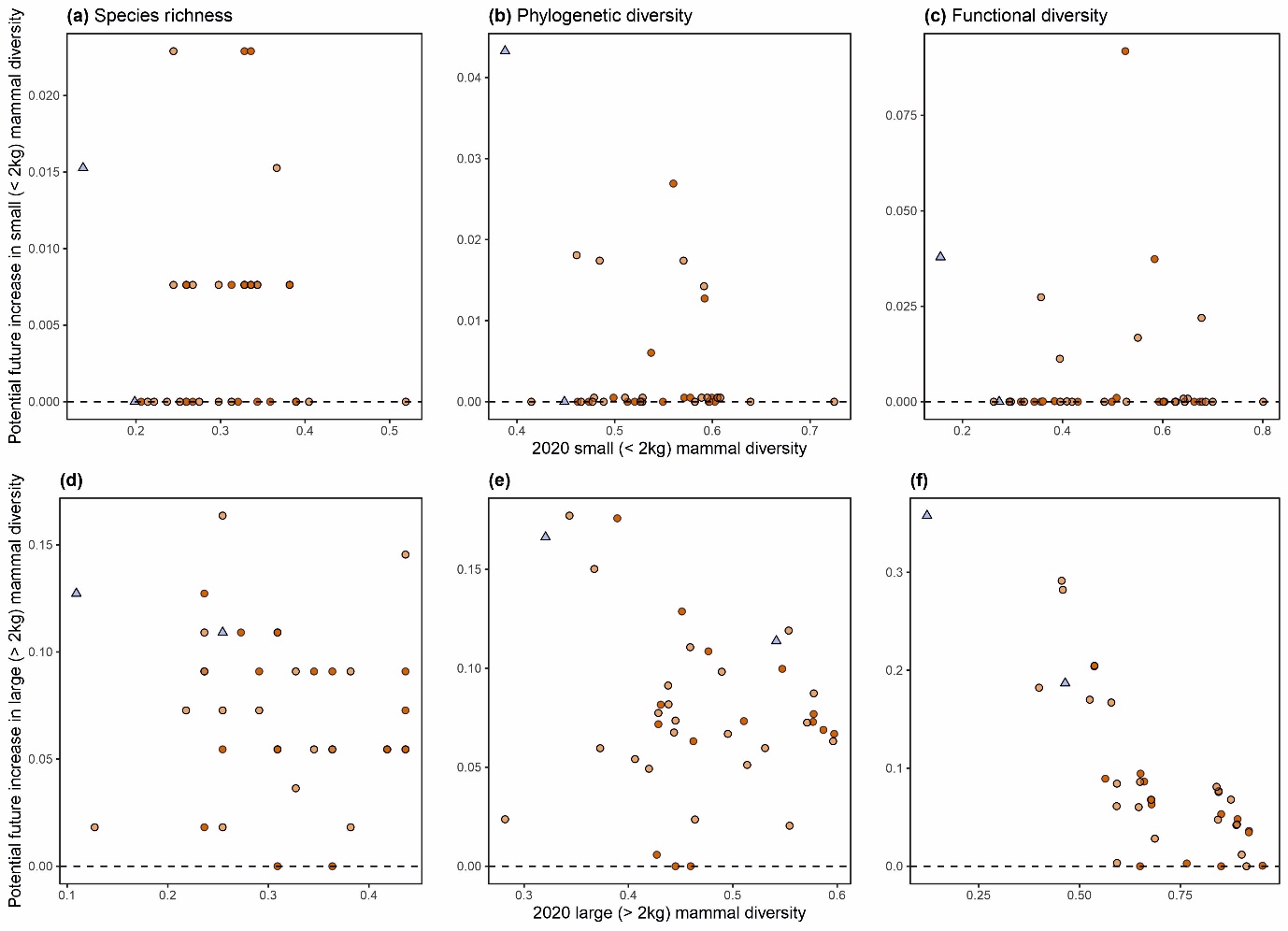


**Fig. S5** Potential future increases on 2020 values in species richness (a and d), phylogenetic diversity (b and e) and functional diversity (c and f) if all reintroductions could be made. Values considering small mammal species (< 2kg and excluding bats) are shown in panels a - c and large mammal species (> 2kg and excluding bats) in panels d -f. Values are scaled within group by the maximum obtainable values (e.g., for small mammals excluding bats, in proportion to the total diversity value for an assemblage containing all small mammals excluding bats). The dashed line represents zero change. Island regions are shown as light blue triangles and continental regions as light brown circles. The Aegean Islands and Crete, the Balearic Islands, Corsica, the island of Ireland, Malta and Sardinia were excluded here as they contained too few species in one or more groups for metric calculation.

**
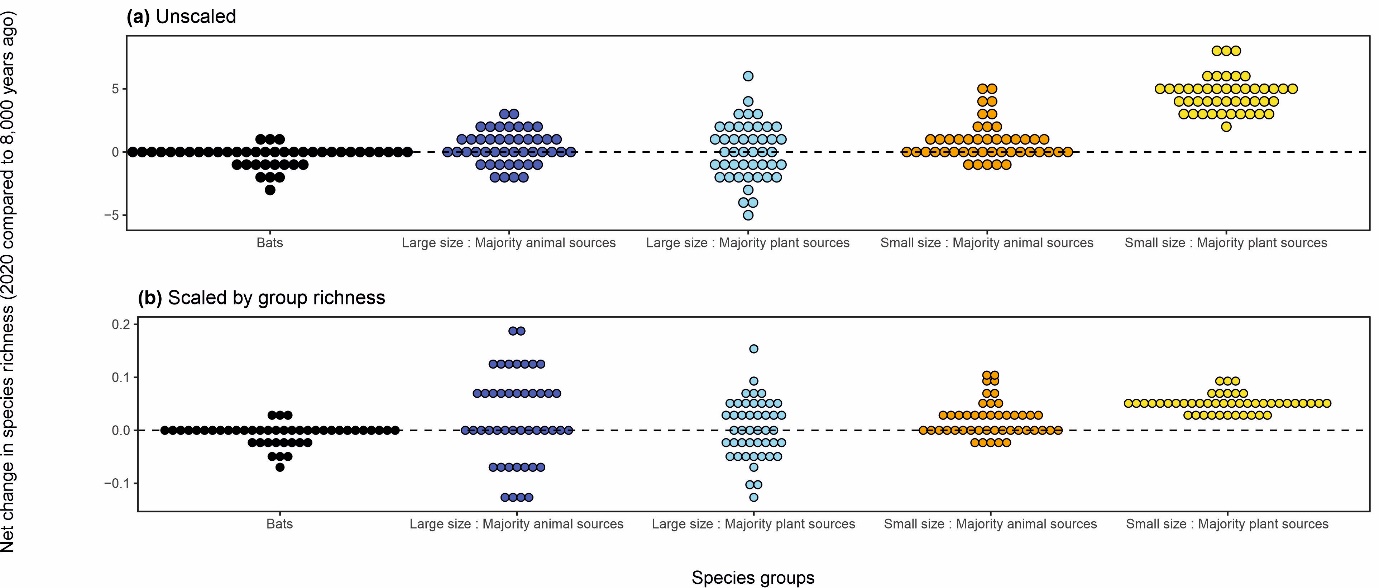
Fig. S6** Net change in species richness (a) and species richness scaled by total group richness (b) comparing 8,000 years ago and 2020. Values considering bats are shown in black, large mammal species (> 2kg) with predominantly animal based diets in dark blue, large mammals with predominantly plant based diets in light blue, small mammals (< 2kg) with predominantly animal based diets in orange and small mammals with predominantly plant based diets in yellow. The dashed line represents zero change. Each dot represents a single region.

**References for supplementary materials**

Abbazzi, L., Angelone, C., Arca, M., Barisone, G., Bedetti, C., Delfino, M., Kotsakis, T., Marcolini, F., Palombo, M. R., Pavia, M., Piras, P., Rook, L., Torre, D., Tuveri, C., Valli, A., & Wilkens, B. (2004). Plio-Pleistocene fossil vertebrates of Monte Tuttavista (Orosei, E. Sardinia, italy), an overview. Rivista Italiana Di Paleontologia e Strartigrafia, 110(3), 681–706.

Alcover, J. A., & Bover, P. (2000). Extreme Insular Evolution in Myotragus balearicus Bate 1909 (Artiodactyla, Caprinae). Tropics, 10(1), 189–201.

Benda, P., Hulva, P., & Gaisler, J. (2004). Systematic status of African populations of Pipistrellus pipistrellus complex (Chiroptera: Vespertilionidae), with a description of a new species from Cyrenaica, Libya. Acta Chiropterologica, 6(2), 193–217.

Bennett, E. A., Champlot, S., Peters, J., Arbuckle, B. S., Guimaraes, S., Pruvost, M., Bar-David, S., Davis, S. J. M., Gautier, M., Kaczensky, P., Kuehn, R., Mashkour, M., Morales-Muñiz, A., Pucher, E., Tournepiche, J.-F., Uerpmann, H.-P., Bălăşescu, A., Germonpré, M., Gündem, C. Y., … Geigl, E.-M. (2017). Taming the late Quaternary phylogeography of the Eurasiatic wild ass through ancient and modern DNA. PLOS ONE, 12(4), 1–28. <https://doi.org/10.1371/journal.pone.0174216>

Baselga, A., Orme, D., Villeger, S., De Bortoli, J., Leprieur, F., & Logez, M. (2021). betapart: Partitioning Beta Diversity into Turnover and Nestedness Components. R package version 1.5.3. https://cran.r-project.org/package=betapart

Moncunill-Solé, B., Jordana, X., & Köhler, M. (2016). How common is gigantism in insular fossil shrews? Examining the ‘Island Rule’ in soricids (Mammalia: Soricomorpha) from Mediterranean Islands using new body mass estimation models. Zoological Journal of the Linnean Society, 178(1), 163–182. https://doi.org/10.1111/zoj.12399

Bover, P., & Alcover, J. A. (2008). Extinction of the autochthonous small mammals of Mallorca (Gymnesic Islands, Western Mediterranean) and its ecological consequences. Journal of Biogeography, 35(6), 1112–1122. [https://doi.org/10.1111/j.1365-2699.2007.01839.x](https://doi.org/https:/doi.org/10.1111/j.1365-2699.2007.01839.x)

Bover, P., Mitchell, K. J., Llamas, B., Rofes, J., Thomson, V. A., Cuenca-Bescós, G., Alcover, J. A., Cooper, A., & Pons, J. (2018). Molecular phylogenetics supports the origin of an endemic Balearic shrew lineage (Nesiotites) coincident with the Messinian Salinity Crisis. Molecular Phylogenetics and Evolution, 125, 188–195. https://doi.org/10.1016/j.ympev.2018.03.028

Bover, P., Llamas, B., Mitchell, K. J., Thomson, V. A., Alcover, J. A., Lalueza-Fox, C., Cooper, A., & Pons, J. (2019). Unraveling the phylogenetic relationships of the extinct bovid Myotragus balearicus Bate 1909 from the Balearic Islands. Quaternary Science Reviews, 215, 185–195. [https://doi.org/10.1016/j.quascirev.2019.05.005](https://doi.org/https:/doi.org/10.1016/j.quascirev.2019.05.005)

Bover, P., Mitchell, K. J., Torres-Roig, E., Llamas, B., Thomson, V. A., Alcover, J. A., Agustí, J., Cooper, A., & Pons, J. (2020). Ancient DNA from an extinct Mediterranean micromammal—Hypnomys morpheus (Rodentia: Gliridae)—Provides insight into the biogeographic history of insular dormice. Journal of Zoological Systematics and Evolutionary Research, 58(1), 427–438. https://doi.org/10.1111/jzs.12343

Chamberlain, S. (2020). rredlist: “IUCN” Red List Client. R package version 0.7.0. https://cran.r-project.org/package=rredlist

Cohen, K.M., Finney, S.C., Gibbard, P.L. & Fan, J.-X. (2013) The ICS International Chronostratigraphic Chart. Episodes 36: 199-204.

Crees, J. J., & Turvey, S. T. (2014). Holocene extinction dynamics of Equus hydruntinus, a late-surviving European megafaunal mammal. Quaternary Science Reviews, 91, 16–29. https://doi.org/10.1016/j.quascirev.2014.03.003

Crees, J. J., Turvey, S. T., Freeman, R., & Carbone, C. (2019). Mammalian tolerance to humans is predicted by body mass: evidence from long-term archives. Ecology, 100(9), e02783. https://doi.org/10.1002/ecy.2783

Cserkész, T., Rusin, M., & Sramkó, G. (2016). An integrative systematic revision of the European southern birch mice (Rodentia: Sminthidae, Sicista subtilis group). Mammal Review, 46(2), 114–130. https://doi.org/10.1111/mam.12058

Cucchi, T., Vigne, J.-D., & Auffray, J.-C. (2005). First occurrence of the house mouse (Mus musculus domesticus Schwarz & Schwarz, 1943) in the Western Mediterranean: a zooarchaeological revision of subfossil occurrences. Biological Journal of the Linnean Society, 84(3), 429–445. <https://doi.org/10.1111/j.1095-8312.2005.00445.x>

Ellis, E. C. (2018). The Great Acceleration. In “The Great Acceleration” in Anthropocene: A Very Short Introduction (Online Edi, pp. 52–77). Oxford University Press.

Faurby, S., & Svenning, J.-C. (2015). A species-level phylogeny of all extant and late Quaternary extinct mammals using a novel heuristic-hierarchical Bayesian approach. Molecular Phylogenetics and Evolution, 84, 14–26. <https://doi.org/10.1016/j.ympev.2014.11.001>

Faurby, S., & Svenning, J.-C. (2016). Resurrection of the Island Rule: Human-Driven Extinctions Have Obscured a Basic Evolutionary Pattern. The American Naturalist, 187(6), 812–820. https://doi.org/10.1086/686268

Faurby, S., Davis, M., Pedersen, R. Ø., Schowanek, S. D., Antonelli, A., & Svenning, J.-C. (2018). PHYLACINE 1.2: the phylogenetic atlas of mammal macroecology. Ecology, 99(11), 2626.

Faurby, S., Pedersen RØand Davis, M., Schowanek, S. D., Jarvie, S., Antonelli, A., & Svenning, J. C. (2020). PHYLACINE 1.2. 1: An Update to the Phylogenetic Atlas of Mammal Macroecology.

Genovesi, P., Bacher, S., Kobelt, M., Pascal, M., & Scalera, R. (2009). Alien Mammals of Europe. In Handbook of Alien Species in Europe. Springer.

Habel, K., Grasman, R., Gramacy, R. B., Mozharovskyi, P., & Sterratt, D. C. (2019). geometry: Mesh Generation and Surface Tessellation. https://cran.r-project.org/package=geometry

Halley, D. J., Saveljev, A. P., & Rosell, F. (2021). Population and distribution of beavers Castor fiber and Castor canadensis in Eurasia. Mammal Review, 51(1), 1–24. <https://doi.org/10.1111/mam.12216>

Hornik, K. (2005). A CLUE for CLUster Ensembles. Journal of Statistical Software, 14(12). <https://doi.org/10.18637/jss.v014.i12>

Hornik, K. (2021). clue: Cluster ensembles. <https://cran.r-project.org/package=clue>

Integrated Taxonomic Information System (ITIS) on-line database, [www.itis.gov](http://www.itis.gov), <https://doi.org/10.5066/F7KH0KBK>. Last accessed 02/2022.

IUCN, The IUCN Red List of Threatened Species. Version 2021-1. (2021a). https://www.iucnredlist.org. Maps downloaded 03/2021.

IUCN, The IUCN Red List of Threatened Species. Version 2021-3. (2021b). <https://www.iucnredlist.org>. Current versions accessed online from 03/2020 to 12/2021.

Juste, J., Benda, P., Garcia-Mudarra, J. L., & Ibáñez, C. (2013). Phylogeny and systematics of Old World serotine bats (genus Eptesicus, Vespertilionidae, Chiroptera): an integrative approach. Zoologica Scripta, 42(5), 441–457. [https://doi.org/10.1111/zsc.12020](https://doi.org/https:/doi.org/10.1111/zsc.12020)

Kay, M. (2021). ggdist*: Visualizations of Distributions and Uncertainty*. https://doi.org/10.5281/zenodo.3879620

Kembel, S. W., Cowan, P. D., Helmus, M. R., Cornwell, W. K., Morlon, H., Ackerly, D. D., Blomberg, S. P., & Webb, C. O. (2010). Picante: R tools for integrating phylogenies and ecology. Bioinformatics, 26, 1463–1464.

Laliberté, E., & Legendre, P. (2010). A distance-based framework for measuring functional diversity from multiple traits. Ecology, 91, 299–305.

Laliberté, E., Legendre, P., & Shipley, B. (2014). FD: measuring functional diversity from multiple traits, and other tools for functional ecology.

Maechler, M., Rousseeuw, P., Struyf, A., Hubert, M., & Hornik, K. (2021). cluster: Cluster Analysis Basics and Extensions. https://cran.r-project.org/package=cluster

McNeill, J. R. and Engelke, P. (2014). *The Great Acceleration: An Environmental History of the Anthropocene since 1945*. Cambridge USA: Bekknap Press of Harvard University Press.

Monastersky, R. (2015). First atomic blast proposed as start of Anthropocene. Nature. <https://doi.org/10.1038/nature.2015.16739>

Naundrup, P. J., & Svenning, J.-C. (2015). A Geographic Assessment of the Global Scope for Rewilding with Wild-Living Horses (Equus ferus). PLOS ONE, 10(7), 1–26. <https://doi.org/10.1371/journal.pone.0132359>

Neuwirth, E. (2014). RColorBrewer: ColorBrewer Palettes. https://cran.r-project.org/package=RColorBrewer

Paradis, E., & Schliep, K. (2019). ape 5.0: an environment for modern phylogenetics and evolutionary analyses in {R}. Bioinformatics, 35, 526–528.

Pebesma, E. (2018). Simple Features for R: Standardized Support for Spatial Vector Data. The R Journal, 10(1), 439–446. <https://doi.org/10.32614/RJ-2018-009>

QGIS Development Team, 2021. QGIS Geographic Information System. QGIS Association. <https://www.qgis.org>.

Raia, P., & Meiri, S. (2006). The island rule in large mammals: paleontology meets ecology. Evolution, 60(8), 1731–1742. [https://doi.org/10.1111/j.0014-3820.2006.tb00516.x](https://doi.org/https:/doi.org/10.1111/j.0014-3820.2006.tb00516.x)

R Core Team, 2021. R: A Language and Environment for Statistical Computing. R Foundation for Statistical Computing: Vienna, Austria. [https://www.R-project.org/](https://www.r-project.org/)

Salotti, M., Bailon, S., Bonifay, M.-F., Courtois, J.-Y., Dubois, J.-N., Ferrandini, J., Ferrandini, M., Milza, J.-C. La, Mourer-Chauviré, C., Popelard, J.-B., Quinif, Y., Réal-Testud, A.-M., Miniconi, C., Pereira, E., Persiani, C., Corse, K., Aead, C. R., & Laboratoire D’anthropologie, F. B. : (1997). Castiglione 3, un nouveau remplissage fossilifère d’âge Pléistocène mojendans le karst de la région d’Oletta (Haute-Corse). Comptes Rendus de L’Académie Des Sciences, Paris, Série B. https://hal-mnhn.archives-ouvertes.fr/mnhn-03262214

Smith, F. A., Lyons, S. K., Ernest, S. K. M., Jones, K. E., Kaufman, D. M., Dayan, T., Marquet, P. A., Brown, J. H., & Haskell, J. P. (2003). Body Mass Of Late Quaternary Mammals. Ecology, 84(12), 3403. [https://doi.org/10.1890/02-9003](https://doi.org/https:/doi.org/10.1890/02-9003)

Smith, W. J., Quilodrán, C. S., Jezierski, M. T., Sendell-Price, A. T., & Clegg, S. M. (2021). The wild ancestors of domestic animals as a neglected and threatened component of biodiversity. Conservation Biology, [https://doi.org/10.1111/cobi.13867](https://doi.org/https:/doi.org/10.1111/cobi.13867).

Traveset, A., Nogales, M., Alcover, J. A., Delgado, J. D., López-Darias, M., Godoy, D., Igual, J. M., & Bover, P. (2009). A review on the effects of alien rodents in the Balearic (Western Mediterranean Sea) and Canary Islands (Eastern Atlantic Ocean). Biological Invasions, 11(7), 1653–1670. https://doi.org/10.1007/s10530-008-9395-y

Turvey, S. T. (2009). Holocene mammal extinctions. Holocene Extinctions, 41–61. Oxford University Press.

Upham, N. S., Esselstyn, J. A., & Jetz, W. (2019). Inferring the mammal tree: Species-level sets of phylogenies for questions in ecology, evolution, and conservation. PLOS Biology, 17(12), 1–44. <https://doi.org/10.1371/journal.pbio.3000494>

van den Hoek Ostende, L. W., van der Geer, A. A. E., & Wijngaarden, C. L. (2017). Why are there no giants at the dwarves feet? Insular micromammals in the eastern Mediterranean. Quaternary International, 445, 269–278. https://doi.org/10.1016/j.quaint.2016.05.007

van der Geer, A. A., Lyras, G. A., Lomolino, M. V, Palombo, M. R., & Sax, D. F. (2013). Body size evolution of palaeo-insular mammals: temporal variations and interspecific interactions. Journal of Biogeography, 40(8), 1440–1450. https://doi.org/10.1111/jbi.12119

Voosen, P. (2016). Anthropocene pinned to postwar period. Science, 353(6302), 852–853. <https://doi.org/10.1126/science.353.6302.852>

Warnes, G. R., Bolker, B., & Lumley, T. (2021). gtools: Various R Programming Tools. https://cran.r-project.org/package=gtools

Wickham, H. (2007). Reshaping Data with the reshape Package. Journal of Statistical Software, 21(12), 1–20.

Wickham, H. (2011). The Split-Apply-Combine Strategy for Data Analysis. Journal of Statistical Software, 40(1), 1–29.

Wickham, H. (2016). ggplot2: Elegant Graphics for Data Analysis. In Springer-Verlag New York. http://ggplot2.org

Wickham, H. (2019). stringr: Simple, Consistent Wrappers for Common String Operations. <https://cran.r-project.org/package=stringr>

Wickham, H., & Seidel, D. (2020). scales: Scale Functions for Visualization. <https://cran.r-project.org/package=scales>

Wickham, H., François, R., Henry, L., & Müller, K. (2021). dplyr: A Grammar of Data Manipulation. https://cran.r-project.org/package=dplyr

Wilke, C. O. (2020). cowplot: Streamlined Plot Theme and Plot Annotations for “ggplot2.” https://cran.r-project.org/package=cowplot

Wilman, H., Belmaker, J., Simpson, J., de la Rosa, C., Rivadeneira, M. M., & Jetz, W. (2014). EltonTraits 1.0: Species-level foraging attributes of the world’s birds and mammals. Ecology, 95(7), 2027. https://doi.org/10.1890/13-1917.1

**Additional Sources for species regional occurrence data compilation**

*References used for all regions*

Crees, J. J., Turvey, S. T., Freeman, R., & Carbone, C. (2019a). Mammalian tolerance to humans is predicted by body mass: evidence from long-term archives. Ecology, 100(9), e02783. https://doi.org/10.1002/ecy.2783

Invasive Species Specialist Group ISSG, The Global Invasive Species Database. Version 2015.1 (2015) http://www.iucngisd.org/gisd/ last accessed 05/2021.

IUCN, The IUCN Red List of Threatened Species. Version 2021-3. (2021). <https://www.iucnredlist.org>. Current versions accessed online from 03/2020 to 12/2021.

Pagad, S., Genovesi, P., Carnevali, L., Schigel, D., & McGeoch, M. A. (2018). Introducing the Global Register of Introduced and Invasive Species. Scientific Data, 5(1), 170202. <https://doi.org/10.1038/sdata.2017.202>

Roy. D., Alderman, D., Anastasiu, P., Arianoutsou, M., Augustin, S., Bacher, S., Başnou, C., Beisel, J., Bertolino, S., Bonesi, L., Bretagnolle, F., Chapuis, J. L., Chauvel, B., Chiron, F., Clergeau, P., Cooper, J., Cunha, T., Delipetrou, P., Desprez-Loustau, M., … Reyserhove, L. (2019). DAISIE - Inventory of alien invasive species in Europe. v1.6. Research Institute for Nature and Forest (INBO). Dataset/Checklist. <https://ipt.inbo.be/resource?r=daisie-checklist&v=1.6> accessed via GBIF.org 03/2020.

Turvey, S. T. (2009a). In the shadow of the megafauna: prehistoric mammal and bird extinctions across the Holocene. Holocene Extinctions, 17- 41.

Turvey, S. T. (2009b). Holocene mammal extinctions. Holocene Extinctions, 41–61.

Turvey, S. T., & Fritz, S. A. (2011). The ghosts of mammals past: biological and geographical patterns of global mammalian extinction across the Holocene. Philosophical Transactions of the Royal Society B: Biological Sciences, 366(1577), 2564–2576. <https://doi.org/10.1098/rstb.2011.0020>

*Additional references used for each region*

Aegean Islands and Crete

Cucchi, T., Papayianni, K., Cersoy, S., Aznar-Cormano, L., Zazzo, A., Debruyne, R., Berthon, R., Bălășescu, A., Simmons, A., Valla, F., Hamilakis, Y., Mavridis, F., Mashkour, M., Darvish, J., Siahsarvi, R., Biglari, F., Petrie, C. A., Weeks, L., Sardari, A., … Vigne, J.-D. (2020). Tracking the Near Eastern origins and European dispersal of the western house mouse. *Scientific Reports*, *10*(1), 8276. https://doi.org/10.1038/s41598-020-64939-9

Dubey, S., Cosson, J.-F., Magnanou, E., Vohralík, V., Benda, P., Frynta, D., Hutterer, R., Vogel, V., & Vogel, P. (2007). Mediterranean populations of the lesser white-toothed shrew (*Crocidura suaveolens* group): an unexpected puzzle of Pleistocene survivors and prehistoric introductions. *Molecular Ecology*, *16*(16), 3438–3452. https://doi.org/10.1111/j.1365-294X.2007.03396.x

Masseti, M. (2012). *Atlas of terrestrial mammals of the Ionian and Aegean islands*. Walter de Gruyter.

Masseti, M., Pecchioli, E., & Vernesi, C. (2008). Phylogeography of the last surviving populations of Rhodian and Anatolian fallow deer (*Dama dama dama* L., 1758). *Biological Journal of the Linnean Society*, *93*(4), 835–844. https://doi.org/10.1111/j.1095-8312.2007.00951.x

Mitchell-Jones, A. J., Amori, G., Bogdanowicz, W., Krystufek, B., Reijnders, P. J. H., Spitzenberger, F., Stubbe, M., Thissen, J. B. M., Vohralik, V., & Zima, J. (1999). *The Atlas of European Mammals*. Academic Press.

Papayiannis, K. (2012). The micromammals of Minoan Crete: human intervention in the ecosystem of the island. *Palaeobiodiversity and Palaeoenvironments*, *92*(2), 239–248. https://doi.org/10.1007/s12549-012-0081-9

Rodrigues, M., Bos, A. R., Schembri, P. J., de Lima, R. F., Lymberakis, P., Parpal, L., Cento, M., Ruette, S., Ozkurt, S. O., Santos-Reis, M., Merilä, J., & Fernandes, C. (2017). Origin and introduction history of the least weasel (Mustela nivalis) on Mediterranean and Atlantic islands inferred from genetic data. *Biological Invasions*, *19*(1), 399–421. https://doi.org/10.1007/s10530-016-1287-y

Theodorou, G., Symeonidis, N., & Stathopoulou, E. (2007). Elephas tiliensis n. sp. from Tilos island (Dodecanese, Greece). *Hellenic Journal of Geosciences*, *42*(1), 19–32.

Zenetos A., Arianoutsou M., Bazos I., Christopoulou A., Kokkoris Y., Zervou S., Zikos A., Wong L.J., & Pagad S. (2020). Global Register of Introduced and Invasive Species - Greece. v1.1. *Invasive Species Specialist Group ISSG*, Dataset/Checklist https://cloud.gbif.org/griis/resource?r=griis-greece&v=1.1 Downloaded via GBIF.org 03/2020.

Albania

Arnold, J., Humer, A., Heltai, M., Murariu, D., Spassov, N., & Hackländer, K. (2012). Current status and distribution of golden jackals Canis aureus in Europe. *Mammal Review*, *42*(1), 1–11. https://doi.org/10.1111/j.1365-2907.2011.00185.x

Genovesi, P., Bacher, S., Kobelt, M., Pascal, M., & Scalera, R. (2009). Alien Mammals of Europe. In *Handbook of Alien Species in Europe*. Springer.

Krofel, M., Giannatos, G., Ćirovič, D., Stoyanov, S., & Newsome, T. M. (2017). Golden jackal expansion in Europe: a case of mesopredator release triggered by continent-wide wolf persecution? *Hystrix, the Italian Journal of Mammalogy*, *28*(1), 9–15. https://doi.org/10.4404/hystrix-28.1-11819

Prigioni, C. (1996). Distribution of mammals in Albania. *Hystrix, the Italian Journal of Mammalogy*, *8*(1–2). https://doi.org/10.4404/hystrix-8.1-2-4095

Rakaj, M., & Pagad, S. (2020). Global Register of Introduced and Invasive Species- Albania. v1.4. *Invasive Species Specialist Group ISSG*, Dataset/Checklist https://cloud.gbif.org/griis/resource?r=griis-albania&v=1.4 Downloaded via GBIF.org 03/2020.

Andorra

 Barrio, I. C., Herrero, J., Bueno, C. G., López, B. C., Aldezabal, A., Campos-Arceiz, A., & García-González, R. (2013). The successful introduction of the alpine marmot Marmota marmota in the Pyrenees, Iberian Peninsula, Western Europe. *Mammal Review*, *43*(2), 142–155. https://doi.org/10.1111/j.1365-2907.2012.00212.x

Gaubert, P., Machordom, A., Morales, A., López-Bao, J. V., Veron, G., Amin, M., Barros, T., Basuony, M., Djagoun, C. A. M. S., San, E. D. L., Fonseca, C., Geffen, E., Ozkurt, S. O., Cruaud, C., Couloux, A., & Palomares, F. (2011). Comparative phylogeography of two African carnivorans presumably introduced into Europe: disentangling natural versus human-mediated dispersal across the Strait of Gibraltar. *Journal of Biogeography*, *38*(2), 341–358. https://doi.org/10.1111/j.1365-2699.2010.02406.x

Mitchell-Jones, A. J., Amori, G., Bogdanowicz, W., Krystufek, B., Reijnders, P. J. H., Spitzenberger, F., Stubbe, M., Thissen, J. B. M., Vohralik, V., & Zima, J. (1999). *The Atlas of European Mammals*. Academic Press.

Salas Sopena, M., Moles, A., Wong, L.J., & Pagad, S. (2020). Global Register of Introduced and Invasive Species - Andorra. v1.5. *Invasive Species Specialist Group ISSG*, Dataset/Checklist https://cloud.gbif.org/griis/resource?r=griis-andorra&v=1.5 Downloaded via GBIF.org 03/2020.

Austria

Arnold, J., Humer, A., Heltai, M., Murariu, D., Spassov, N., & Hackländer, K. (2012). Current status and distribution of golden jackals Canis aureus in Europe. *Mammal Review*, *42*(1), 1–11. https://doi.org/10.1111/j.1365-2907.2011.00185.x

Crees, J. J., & Turvey, S. T. (2014). Holocene extinction dynamics of Equus hydruntinus, a late-surviving European megafaunal mammal. *Quaternary Science Reviews*, *91*, 16–29. https://doi.org/10.1016/j.quascirev.2014.03.003

Essl, F., Rabitsch, W., Wong, L.J. & Pagad, S. (2020). Global Register of Introduced and Invasive Species- Austria. v1.3. *Invasive Species Specialist Group ISSG*, Dataset/Checklist https://cloud.gbif.org/griis/resource?r=griis-austria&v=1.3 Downloaded via GBIF.org 03/2020.

Genovesi, P., Bacher, S., Kobelt, M., Pascal, M., & Scalera, R. (2009). Alien Mammals of Europe. In *Handbook of Alien Species in Europe*. Springer.

Krofel, M., Giannatos, G., Ćirovič, D., Stoyanov, S., & Newsome, T. M. (2017). Golden jackal expansion in Europe: a case of mesopredator release triggered by continent-wide wolf persecution? *Hystrix, the Italian Journal of Mammalogy*, *28*(1), 9–15. https://doi.org/10.4404/hystrix-28.1-11819

Maran, T. (2007). *Conservation biology of the European mink, Mustela lutreola (Linnaeus 1761): decline and causes of extinction*. Tallinn University.

Nolet, B. A., & Rosell, F. (1998). Comeback of the beaver *Castor fiber*: An overview of old and new conservation problems. *Biological Conservation*, *83*(2), 165–173. https://doi.org/https://doi.org/10.1016/S0006-3207(97)00066-9

Balearic Islands

 Alcover, J. A. (2010). Introduccions de mamífers a les Balears: l’establiment d’un nou ordre. In *Seminari sobre espècies introduïdes i invasores a les Illes Balears*, 175–186.

Bover, P., & Alcover, J. A. (2008). Extinction of the autochthonous small mammals of Mallorca (Gymnesic Islands, Western Mediterranean) and its ecological consequences. *Journal of Biogeography*, *35*(6), 1112–1122. https://doi.org/10.1111/j.1365-2699.2007.01839.x

Bover, P., Parpal, L., Pons, J., & Alcover, J. A. (2012). Evidence for a recent introduction of Crocidura russula (Mammalia, Eulipotyphla, Soricomorpha) in Mallorca (Balearic Islands, western Mediterranean Sea). *Mammalia*, *76*(1), 113–116. https://doi.org/https://doi.org/10.1515/mamm.2011.101

Cucchi, T., Vigne, J.-D., & Auffray, J.-C. (2005). First occurrence of the house mouse (Mus musculus domesticus Schwarz & Schwarz, 1943) in the Western Mediterranean: a zooarchaeological revision of subfossil occurrences. *Biological Journal of the Linnean Society*, *84*(3), 429–445. https://doi.org/10.1111/j.1095-8312.2005.00445.x

Dana, E. D., García-Berthou, E., Wong, L. J., & Pagad, S. (2020). Global Register of Introduced and Invasive Species- Spain. v1.1. *Invasive Species Specialist Group ISSG*, Dataset/Checklist https://cloud.gbif.org/griis/resource?r=griis-spain&v=1.1 Downloaded via GBIF.org 03/2020.

Gaubert, P., Machordom, A., Morales, A., López-Bao, J. V., Veron, G., Amin, M., Barros, T., Basuony, M., Djagoun, C. A. M. S., San, E. D. L., Fonseca, C., Geffen, E., Ozkurt, S. O., Cruaud, C., Couloux, A., & Palomares, F. (2011). Comparative phylogeography of two African carnivorans presumably introduced into Europe: disentangling natural versus human-mediated dispersal across the Strait of Gibraltar. *Journal of Biogeography*, *38*(2), 341–358. https://doi.org/10.1111/j.1365-2699.2010.02406.x

Khaldi, M., Ribas, A., Barech, G., Hugot, J.-P., Benyettou, M., Albane, L., Arrizabalaga, A., & Nicolas, V. (2016). Molecular evidence supports recent anthropogenic introduction of the Algerian hedgehog Atelerix algirus in Spain, Balearic and Canary islands from North Africa. *Mammalia*, *80*(3), 313–320. https://doi.org/10.1515/mammalia-2014-0180

Pinya, S., & Lassnig, N. (2018). First record of free-ranging fallow deer (Dama dama) in Mallorca (Balearic Islands, Spain). *Galemys*, *30*, 63–65.

Rodrigues, M., Bos, A. R., Schembri, P. J., de Lima, R. F., Lymberakis, P., Parpal, L., Cento, M., Ruette, S., Ozkurt, S. O., Santos-Reis, M., Merilä, J., & Fernandes, C. (2017). Origin and introduction history of the least weasel (Mustela nivalis) on Mediterranean and Atlantic islands inferred from genetic data. *Biological Invasions*, *19*(1), 399–421. https://doi.org/10.1007/s10530-016-1287-y

Ruffino, L., & Vidal, E. (2010). Early colonization of Mediterranean islands by *Rattus rattus*: a review of zooarcheological data. *Biological Invasions*, *12*(8), 2389–2394. https://doi.org/10.1007/s10530-009-9681-3

Valenzuela, A., & Alcover, J. A. (2015). The chronology of the introduction of two species of Martes (Carnivora, Mustelidae) on the Western Mediterranean Islands: first direct radiocarbon evidence. *Biological Invasions*, *17*(11), 3093–3100. https://doi.org/10.1007/s10530-015-0947-7

Belgium

Desmet, P., Reyserhove, L., Oldoni, D., Groom, Q., Adriaens, T., Vanderhoeven, S., & Pagad, S. (2020). Global Register of Introduced and Invasive Species - Belgium. v1.8. *Invasive Species Specialist Group ISSG*, Dataset/Checklist https://doi.org/10.15468/xoidmd Downloaded via GBIF.org 03/2020.

Genovesi, P., Bacher, S., Kobelt, M., Pascal, M., & Scalera, R. (2009). Alien Mammals of Europe. In *Handbook of Alien Species in Europe*. Springer.

Kervyn, T., Lamotte, S., Nyssen, P., & Verschuren, J. (2009). Major decline of bat abundance and diversity during the last 50 years in southern Belgium. *Belgian Journal of Zoology*, *139*, 124–132.

Nolet, B. A., & Rosell, F. (1998). Comeback of the beaver *Castor fiber*: An overview of old and new conservation problems. *Biological Conservation*, *83*(2), 165–173. https://doi.org/10.1016/S0006-3207(97)00066-9

Bulgaria

Carter, J., & Leonard, B. (2002). A Review of the Literature on the Worldwide Distribution, Spread of, and Efforts to Eradicate the Coypu (Myocastor coypus). *Wildlife Society Bulletin*, *30*(1), 162–175.

Crees, J. J., & Turvey, S. T. (2014). Holocene extinction dynamics of *Equus hydruntinus*, a late-surviving European megafaunal mammal. *Quaternary Science Reviews*, *91*, 16–29. https://doi.org/10.1016/j.quascirev.2014.03.003

Genovesi, P., Bacher, S., Kobelt, M., Pascal, M., & Scalera, R. (2009). Alien Mammals of Europe. In *Handbook of Alien Species in Europe*. Springer.

Maran, T. (2007). *Conservation biology of the European mink, Mustela lutreola (Linnaeus 1761): decline and causes of extinction*. Tallinn University.

Pagad, S., & Wong, L. J. (2020). Global Register of Introduced and Invasive Species – Bulgaria. v1.0. *Invasive Species Specialist Group ISSG*, Dataset/Checklist https://cloud.gbif.org/griis/resource?r=griis-bulgaria&v=1.0 Downloaded via GBIF.org 04/2020.

Bosnia and Herzegovina

Arnold, J., Humer, A., Heltai, M., Murariu, D., Spassov, N., & Hackländer, K. (2012). Current status and distribution of golden jackals Canis aureus in Europe. *Mammal Review*, *42*(1), 1–11. https://doi.org/10.1111/j.1365-2907.2011.00185.x

Genovesi, P., Bacher, S., Kobelt, M., Pascal, M., & Scalera, R. (2009). Alien Mammals of Europe. In *Handbook of Alien Species in Europe*. Springer.

Krofel, M., Giannatos, G., Ćirovič, D., Stoyanov, S., & Newsome, T. M. (2017). Golden jackal expansion in Europe: a case of mesopredator release triggered by continent-wide wolf persecution? *Hystrix, the Italian Journal of Mammalogy*, *28*(1), 9–15. https://doi.org/10.4404/hystrix-28.1-11819

Maslo, S., Wong, L. J., & Pagad, S. (2020). GRIIS Checklist of Introduced and Invasive Species - Bosnia and Herzegovina. v1.2. *Invasive Species Specialist Group ISSG*, Dataset/Checklist. https://cloud.gbif.org/griis/resource?r=griis-bosnia-and-herzegovina&v=1.2 Downloaded via GBIF.org 03/2020.

Trbojević, I., & Trbojević, T. (2016). Distribution and population growth of Eurasian beaver (Castor fiber Linnaeus, 1758) in Bosnia and Herzegovina 10 years after reintroduction. *ГЛАСНИК ШУМАРСКОГ ФАКУЛТЕТА УНИВЕРЗИТЕТА У БАЊОЈ ЛУЦИ*, *1*(25).

Belarus

Genovesi, P., Bacher, S., Kobelt, M., Pascal, M., & Scalera, R. (2009). Alien Mammals of Europe. In *Handbook of Alien Species in Europe*. Springer.

Kauhala, K., & Kowalczyk, R. (2011). Invasion of the raccoon dog *Nyctereutes procyonoides* in Europe: History of colonization, features behind its success, and threats to native fauna. *Current Zoology*, *57*(5), 584–598. https://doi.org/10.1093/czoolo/57.5.584

 Kowalczyk, R., Krasińska, M., Kamiński, T., Górny, M., Struś, P., Hofman-Kamińska, E., & Krasiński, Z. A. (2013). Movements of European bison (Bison bonasus) beyond the Białowieża Forest (NE Poland): range expansion or partial migrations? *Acta Theriologica*, *58*(4), 391–401.

Kurhinen, J., Kulebyakina, E., Zadiraka, E., Mamontov, V., Muravskaya, E., & Hanski, I. K. (2011). Distribution of the Siberian flying squirrel (Pteromys volans L.) in taiga isthmuses between Baltic and White Sea regions. *Acta Zoologica Lituanica*, *21*(4), 306–310.

Maran, T. (2007). *Conservation biology of the European mink, Mustela lutreola (Linnaeus 1761): decline and causes of extinction*. Tallinn University.

Semenchenko, V., Wong, L. J., & Pagad, S. (2020). Global Register of Introduced and Invasive Species- Belarus. v1.3. *Invasive Species Specialist Group ISSG*, Dataset/Checklist https://cloud.gbif.org/griis/resource?r=griis-belarus&v=1.3 Downloaded via GBIF.org 03/2020.

Corsica

 Pietri, C., Alves, P. C., & Melo-Ferreira, J. (2011). Hares in Corsica: high prevalence of *Lepus corsicanus* and hybridization with introduced *L. europaeus* and *L. granatensis*. *European Journal of Wildlife Research*, *57*(2), 313–321. https://doi.org/10.1007/s10344-010-0430-9

Thevenot, J., Albert, A., Collas, M., De Massary, J., Dupont, P., Masse, C., Moutou, F., Poulet, N., Roques, A., Souty-Grosset, C., Vincent, B., Wong, L. J., & Pagad, S. (2020). Global Register of Introduced and Invasive Species- France. v1.2. *Invasive Species Specialist Group ISSG*, Dataset/Checklist https://cloud.gbif.org/griis/resource?r=griis-france&v=1.2 Downloaded via GBIF.org 03/2020.

 Vigne, J.-D. (1992). Zooarchaeology and the biogeographical history of the mammals of Corsica and Sardinia since the last ice age. *Mammal Review*, *22*(2), 87–96. https://doi.org/10.1111/j.1365-2907.1992.tb00124.x

Croatia

Boršić, I., Kutleša, P., Desnica, S., Bošnjak, D., Slivar, S., Wong L. J., & Pagad, S. (2017). Global Register of Introduced and Invasive Species- Croatia. v2.4. *Invasive Species Specialist Group ISSG*, Dataset/Checklist http://doi.org/10.15468/rhmen3 Downloaded via GBIF.org 03/2020.

 Genovesi, P., Bacher, S., Kobelt, M., Pascal, M., & Scalera, R. (2009). Alien Mammals of Europe. In *Handbook of Alien Species in Europe*. Springer.

Halley, D. J., Saveljev, A. P., & Rosell, F. (2021). Population and distribution of beavers *Castor fiber* and *Castor canadensis* in Eurasia. *Mammal Review*, *51*(1), 1–24. https://doi.org/10.1111/mam.12216

 Maran, T. (2007). *Conservation biology of the European mink, Mustela lutreola (Linnaeus 1761): decline and causes of extinction*. Tallinn University.

Nelson, M. E. (1993). Natal Dispersal and Gene Flow in White-Tailed Deer in Northeastern Minnesota. *Journal of Mammalogy*, *74*(2), 316–322. https://doi.org/10.2307/1382386

Purger, J., & Kryštufek, B. (1991). Feral Coypu Myocastor coypus (Rodentia, Mammalia) in Yugoslavia. *BIOLOSKI VESTNIK*, *39*(4), 19–24.

Tvrtković, N. (2016). The findings of Mehely’s horseshoe bat (Chiroptera) in Croatia in the last century were mistakes in identification. *Natura Croatica: Periodicum Musei Historiae Naturalis Croatici*, *25*(1), 165–172.

Czechia

 Crees, J. J., & Turvey, S. T. (2014). Holocene extinction dynamics of *Equus hydruntinus*, a late-surviving European megafaunal mammal. *Quaternary Science Reviews*, *91*, 16–29. https://doi.org/10.1016/j.quascirev.2014.03.003

Dvořák, S., Barták, V., Macháček, Z., & Matějů, J. (2014). Home range size and spatio-temporal dynamics of male sika deer (*Cervus nippon*; Cervidae, Artiodactyla) in an introduced population. *Folia Zoologica*, *63*(2), 103–115. https://doi.org/10.25225/fozo.v63.i2.a8.2014

Genovesi, P., Bacher, S., Kobelt, M., Pascal, M., & Scalera, R. (2009). Alien Mammals of Europe. In *Handbook of Alien Species in Europe*. Springer.

Maran, T. (2007). *Conservation biology of the European mink, Mustela lutreola (Linnaeus 1761): decline and causes of extinction*. Tallinn University.

Nolet, B. A., & Rosell, F. (1998). Comeback of the beaver *Castor fiber*: An overview of old and new conservation problems. *Biological Conservation*, *83*(2), 165–173. https://doi.org/10.1016/S0006-3207(97)00066-9

Pergl, J., Wong, L. J., & Pagad, S. (2020). Global Register of Introduced and Invasive Species- Czechia. v1.1. *Invasive Species Specialist Group ISSG*, Dataset/Checklist https://cloud.gbif.org/griis/resource?r=griis-czech-republic&v=1.1 Downloaded via GBIF.org 03/2020.

Uhrin, M., Hüttmeir, U., Kipson, M., Estók, P., Sachanowicz, K., Bücs, S., Karapandža, B., Paunović, M., Presetnik, P., & Bashta, A. (2016). Status of Savi’s pipistrelle *Hypsugo savii* (Chiroptera) and range expansion in Central and south‐eastern Europe: a review. *Mammal Review*, *46*(1), 1–16.

Schmölcke, U., & Zachos, F. E. (2005). Holocene distribution and extinction of the moose (*Alces alces*, Cervidae) in Central Europe. *Mammalian Biology*, *70*(6), 329–344.

Denmark

Genovesi, P., Bacher, S., Kobelt, M., Pascal, M., & Scalera, R. (2009). Alien Mammals of Europe. In *Handbook of Alien Species in Europe*. Springer.

Hofman-Kamińska, E., Bocherens, H., Drucker, D. G., Fyfe, R. M., Gumiński, W., Makowiecki, D., Pacher, M., Piličiauskienė, G., Samojlik, T., Woodbridge, J., & Kowalczyk, R. (2019). Adapt or die—Response of large herbivores to environmental changes in Europe during the Holocene. *Global Change Biology*, *25*(9), 2915–2930. https://doi.org/10.1111/gcb.14733

Møller, J., Wong, L. J., & Pagad, S. (2020). Global Register of Introduced and Invasive Species- Denmark. v1.3. *Invasive Species Specialist Group ISSG*, Dataset/Checklist https://cloud.gbif.org/griis/resource?r=griis-denmark&v=1.3 Downloaded via GBIF.org 03/2020.

Nolet, B. A., & Rosell, F. (1998). Comeback of the beaver *Castor fiber*: An overview of old and new conservation problems. *Biological Conservation*, *83*(2), 165–173. https://doi.org/10.1016/S0006-3207(97)00066-9

Estonia

Andersone-Lilley, Ž., Balciauskas, L., Ozolinš, J., Randveer, T., & Tõnisson, J. (2010). Ungulates and their management in the Baltics (Estonia, Latvia and Lithuania). In M. Apollonio, R. Andersen, & R. Putman (Eds.), *European ungulates and their management in the 21st century* (pp. 103–128). Cambridge University Press.

Chapman, N. G., & Chapman, D. I. (1980). The distribution of fallow deer: a worldwide review. *Mammal Review*, *10*(2‐3), 61–138. https://doi.org/10.1111/j.1365-2907.1980.tb00234.x

Genovesi, P., Bacher, S., Kobelt, M., Pascal, M., & Scalera, R. (2009). Alien Mammals of Europe. In *Handbook of Alien Species in Europe*. Springer.

Nolet, B. A., & Rosell, F. (1998). Comeback of the beaver *Castor fiber*: An overview of old and new conservation problems. *Biological Conservation*, *83*(2), 165–173. https://doi.org/10.1016/S0006-3207(97)00066-9

Veeroja, R., & Männil, P. (2013). Population development and reproduction of wild boar (Sus scrofa) in Estonia. *Wildlife Biology in Practice*, *10*(3), 17–21.

 Vunk, E., Wong, L. J., & Pagad, S. (2020). Global Register of Introduced and Invasive Species- Estonia. v1.1. *Invasive Species Specialist Group ISSG*, Dataset/Checklist https://cloud.gbif.org/griis/resource?r=griis-estonia&v=1.1 Downloaded via GBIF.org 03/2020.

Finland

Chapman, N. G., & Chapman, D. I. (1980). The distribution of fallow deer: a worldwide review. *Mammal Review*, *10*(2‐3), 61–138. https://doi.org/10.1111/j.1365-2907.1980.tb00234.x

Genovesi, P., Bacher, S., Kobelt, M., Pascal, M., & Scalera, R. (2009). Alien Mammals of Europe. In *Handbook of Alien Species in Europe*. Springer.

Niemivuo-Lahti, J., Wong, L. J., & Pagad, S. (2020). Global Register of Introduced and Invasive Species- Finland. v1.1. *Invasive Species Specialist Group ISSG*, Dataset/Checklist https://cloud.gbif.org/griis/resource?r=griis-finland&v=1.1 Downloaded via GBIF.org 03/2020.

Nolet, B. A., & Rosell, F. (1998). Comeback of the beaver *Castor fiber*: An overview of old and new conservation problems. *Biological Conservation*, *83*(2), 165–173. https://doi.org/10.1016/S0006-3207(97)00066-9

Maran, T. (2007). *Conservation biology of the European mink, Mustela lutreola (Linnaeus 1761): decline and causes of extinction*. Tallinn University.

France

Brambilla, A., Von Hardenberg, A., Nelli, L., & Bassano, B. (2020). Distribution, status, and recent population dynamics of Alpine ibex Capra ibex in Europe. *Mammal Review*, *50*(3), 267–277. https://doi.org/10.1111/mam.12194

Crees, J. J., & Turvey, S. T. (2014). Holocene extinction dynamics of *Equus hydruntinus*, a late-surviving European megafaunal mammal. *Quaternary Science Reviews*, *91*, 16–29. https://doi.org/10.1016/j.quascirev.2014.03.003

Genovesi, P., Bacher, S., Kobelt, M., Pascal, M., & Scalera, R. (2009). Alien Mammals of Europe. In *Handbook of Alien Species in Europe*. Springer.

Thevenot, J., Albert, A., Collas, M., De Massary, J., Dupont, P., Masse, C., Moutou, F., Poulet, N., Roques, A., Souty-Grosset, C., Vincent, B., Wong, L. J., & Pagad, S. (2020). Global Register of Introduced and Invasive Species- France. v1.2. *Invasive Species Specialist Group ISSG*, Dataset/Checklist https://cloud.gbif.org/griis/resource?r=griis-france&v=1.2 Downloaded via GBIF.org 03/2020.

Great Britain

Crawley, D., Coomber, F., Kubasiewicz, L., Harrower, C., Evans, P., Waggitt, J., Smith, B., & Matthews, F. (Eds.). (2020). *Atlas of the mammals of Great Britain and Northern Ireland*. Pelagic Publishing Ltd.

Genovesi, P., Bacher, S., Kobelt, M., Pascal, M., & Scalera, R. (2009). Alien Mammals of Europe. In *Handbook of Alien Species in Europe*. Springer.

Roy, H., Rorke, S., Wong, L. J., & Pagad, S. (2020). Global Register of Introduced and Invasive Species- Great Britain. v1.5. *Invasive Species Specialist Group ISSG,* Dataset/Checklist https://cloud.gbif.org/griis/resource?r=griis-united_kingdom&v=1.5 Downloaded via GBIF.org 03/2020.

Germany

Bonesi, L., & Palazon, S. (2007). The American mink in Europe: Status, impacts, and control. *Biological Conservation*, *134*(4), 470–483. https://doi.org/10.1016/j.biocon.2006.09.006

Genovesi, P., Bacher, S., Kobelt, M., Pascal, M., & Scalera, R. (2009). Alien Mammals of Europe. In *Handbook of Alien Species in Europe*. Springer.

Gollasch, S., Kühn, I., Wong, L. J., & Pagad, S. (2020). Global Register of Introduced and Invasive Species- Germany. v1.1. *Invasive Species Specialist Group ISSG,* Dataset/Checklist https://cloud.gbif.org/griis/resource?r=griis-germany&v=1.1 Downloaded via GBIF.org 03/2020.

Maran, T. (2007). *Conservation biology of the European mink, Mustela lutreola (Linnaeus 1761): decline and causes of extinction*. Tallinn University.

Matějů, J., Říčanová, Š., Ambros, M., Kala, B., Hapl, E., & Matějů, K. (2010). Reintroductions of the European Ground Squirrel (*Spermophilus citellus*) in Central Europe (Rodentia: Sciuridae). *Lynx, Series Nova*, *41*(1).

Schönfeld, F. (2009). Presence of moose (*Alces alces*) in Southeastern Germany. *European Journal of Wildlife Research*, *55*(4), 449–453.

Uhrin, M., Hüttmeir, U., Kipson, M., Estók, P., Sachanowicz, K., Bücs, S., Karapandža, B., Paunović, M., Presetnik, P., & Bashta, A. (2016). Status of Savi’s pipistrelle *Hypsugo savii* (Chiroptera) and range expansion in Central and south‐eastern Europe: a review. *Mammal Review*, *46*(1), 1–16.

Greece

Breitenmoser-Wuersten, C., & Breitenmoser, U. (2001). The lynx in the Balkans - a summary of present knowledge. *KORA Bericht*, *7*, 32–35.

Carter, J., & Leonard, B. (2002). A Review of the Literature on the Worldwide Distribution, Spread of, and Efforts to Eradicate the Coypu (Myocastor coypus). *Wildlife Society Bulletin*, *30*(1), 162–175.

Cucchi, T., Papayianni, K., Cersoy, S., Aznar-Cormano, L., Zazzo, A., Debruyne, R., Berthon, R., Bălășescu, A., Simmons, A., Valla, F., Hamilakis, Y., Mavridis, F., Mashkour, M., Darvish, J., Siahsarvi, R., Biglari, F., Petrie, C. A., Weeks, L., Sardari, A., … Vigne, J.-D. (2020). Tracking the Near Eastern origins and European dispersal of the western house mouse. *Scientific Reports*, *10*(1), 8276. https://doi.org/10.1038/s41598-020-64939-9

Zenetos, A., Arianoutsou, M., Bazos, I., Christopoulou, A., Kokkoris, Y., Zervou, S., Zikos, A., Wong, L. J., & Pagad, S. (2020). Global Register of Introduced and Invasive Species- Greece. v1.1. *Invasive Species Specialist Group ISSG,* Dataset/Checklist https://cloud.gbif.org/griis/resource?r=griis-greece&v=1.1 Downloaded via GBIF.org 03/2020.

Hungary

Arnold, J., Humer, A., Heltai, M., Murariu, D., Spassov, N., & Hackländer, K. (2012). Current status and distribution of golden jackals Canis aureus in Europe. *Mammal Review*, *42*(1), 1–11. https://doi.org/10.1111/j.1365-2907.2011.00185.x

Borza, P., Anikó, C., Balogh, L., Andras, W., Wong, L. J., & Pagad, S. (2020). Global Register of Introduced and Invasive Species- Hungary. v1.2. *Invasive Species Specialist Group ISSG,* Dataset/Checklist https://cloud.gbif.org/griis/resource?r=griis-hungary&v=1.2 Downloaded via GBIF.org 03/2020.

Crees, J. J., & Turvey, S. T. (2014). Holocene extinction dynamics of *Equus hydruntinus*, a late-surviving European megafaunal mammal. *Quaternary Science Reviews*, *91*, 16–29. https://doi.org/10.1016/j.quascirev.2014.03.003

Demeter, A. (1984). Recent records of rare or non-resident large carnivores in Hungary. *Vertebrata Hungarica*, *22*, 65–71.

Genovesi, P., Bacher, S., Kobelt, M., Pascal, M., & Scalera, R. (2009). Alien Mammals of Europe. In *Handbook of Alien Species in Europe*. Springer.

Halley, D. J., Saveljev, A. P., & Rosell, F. (2021). Population and distribution of beavers *Castor fiber* and *Castor canadensis* in Eurasia. *Mammal Review*, *51*(1), 1–24. https://doi.org/10.1111/mam.12216

Krofel, M., Giannatos, G., Ćirovič, D., Stoyanov, S., & Newsome, T. M. (2017). Golden jackal expansion in Europe: a case of mesopredator release triggered by continent-wide wolf persecution? *Hystrix, the Italian Journal of Mammalogy*, *28*(1), 9–15. https://doi.org/10.4404/hystrix-28.1-11819

Maran, T. (2007). *Conservation biology of the European mink, Mustela lutreola (Linnaeus 1761): decline and causes of extinction*. Tallinn University.

Mitchell-Jones, A. J., Amori, G., Bogdanowicz, W., Krystufek, B., Reijnders, P. J. H., Spitzenberger, F., Stubbe, M., Thissen, J. B. M., Vohralik, V., & Zima, J. (1999). *The Atlas of European Mammals*. Academic Press.

Uhrin, M., Hüttmeir, U., Kipson, M., Estók, P., Sachanowicz, K., Bücs, S., Karapandža, B., Paunović, M., Presetnik, P., & Bashta, A. (2016). Status of Savi’s pipistrelle *Hypsugo savii* (Chiroptera) and range expansion in Central and south‐eastern Europe: a review. *Mammal Review*, *46*(1), 1–16.

Varga, I. (1996). Report on Invasive Alien Species in Hungary. Ministry of Agriculture Hungary.

Island of Ireland

Crawley, D., Coomber, F., Kubasiewicz, L., Harrower, C., Evans, P., Waggitt, J., Smith, B., & Matthews, F. (Eds.). (2020). *Atlas of the mammals of Great Britain and Northern Ireland*. Pelagic Publishing Ltd.

O'Flynn C., O’Callaghan, R., Wong, L. J., & Pagad, S. (2017). Global Register of Introduced and Invasive Species- Ireland. v2.4. *Invasive Species Specialist Group ISSG*, Dataset/Checklist http://doi.org/10.15468/br8rgw Downloaded via GBIF.org 03/2020.

Herman, J. S., Jóhannesdóttir, F., Jones, E. P., McDevitt, A. D., Michaux, J. R., White, T. A., Wójcik, J. M., & Searle, J. B. (2017). Post-glacial colonization of Europe by the wood mouse, *Apodemus sylvaticus*: evidence of a northern refugium and dispersal with humans. *Biological Journal of the Linnean Society*, *120*(2), 313–332. https://doi.org/10.1111/bij.12882

Koprowski, J. L. (1994). Sciurus carolinensis. *Mammalian Species*, *480*, 1–9. https://doi.org/10.2307/3504224

McDevitt, A. D., Carden, R. F., Coscia, I., & Frantz, A. C. (2013). Are wild boars roaming Ireland once more? *European Journal of Wildlife Research*, *59*(5), 761–764. https://doi.org/10.1007/s10344-013-0721-z

McDevitt, A. D., Rambau, R. V, O’Brien, J., McDevitt, C. D., Hayden, T. J., & Searle, J. B. (2009). Genetic variation in Irish pygmy shrews *Sorex minutus* (Soricomorpha: Soricidae): implications for colonization history. *Biological Journal of the Linnean Society*, *97*(4), 918–927. https://doi.org/10.1111/j.1095-8312.2009.01218.x

Roy, H., Rorke, S., Wong, L. J., & Pagad, S. (2020). Global Register of Introduced and Invasive Species- Great Britain. v1.5. *Invasive Species Specialist Group ISSG,* Dataset/Checklist https://cloud.gbif.org/griis/resource?r=griis-united_kingdom&v=1.5 Downloaded via GBIF.org 03/2020.

Searle, J. B. (2008). The colonization of Ireland by mammals. *The Irish Naturalists’ Journal*, *29*, 109–115.

Sheehy, E., & Lawton, C. (2015). Distribution of the non-native Hazel Dormouse (*Muscardinus avellanarius*) in Ireland. *Irish Naturalists’ Journal*, *34*, 13–16.

Tosh, D. G., Lusby, J., Montgomery, W. I. A. N., & O’Halloran, J. (2008). First record of greater white-toothed shrew *Crocidura russula* in Ireland. *Mammal Review*, *38*(4), 321–326. https://doi.org/10.1111/j.1365-2907.2008.00130.x

Italy

Arnold, J., Humer, A., Heltai, M., Murariu, D., Spassov, N., & Hackländer, K. (2012). Current status and distribution of golden jackals Canis aureus in Europe. *Mammal Review*, *42*(1), 1–11. https://doi.org/10.1111/j.1365-2907.2011.00185.x

Carnevali, L., Marchini, A., Occhipinti, A., Genovesi, P., Wong, L. J., & Pagad, S. (2020). Global Register of Introduced and Invasive Species- Italy. v1.4. *Invasive Species Specialist Group ISSG,* Dataset/Checklist https://cloud.gbif.org/griis/resource?r=griis_italy&v=1.4 Downloaded via GBIF.org 03/2020.

Crees, J. J., & Turvey, S. T. (2014). Holocene extinction dynamics of *Equus hydruntinus*, a late-surviving European megafaunal mammal. *Quaternary Science Reviews*, *91*, 16–29. https://doi.org/10.1016/j.quascirev.2014.03.003

Dodaro, G., Battisti, C., Campedelli, T., Fanelli, G., & Monaco, A. (2019). Unsafe management of a zoological garden as a cause of introduction of an alien species into the wild: First documented case of feral naturalized population of Lama glama in Europe. *Journal for Nature Conservation*, *49*, 22–26. https://doi.org/10.1016/j.jnc.2019.02.006

Genovesi, P., Bacher, S., Kobelt, M., Pascal, M., & Scalera, R. (2009). Alien Mammals of Europe. In *Handbook of Alien Species in Europe*. Springer.

Halley, D. J., Saveljev, A. P., & Rosell, F. (2021). Population and distribution of beavers *Castor fiber* and *Castor canadensis* in Eurasia. *Mammal Review*, *51*(1), 1–24. https://doi.org/10.1111/mam.12216

Imperio, S., Focardi, S., Santini, G., & Provenzale, A. (2012). Population dynamics in a guild of four Mediterranean ungulates: density-dependence, environmental effects and inter-specific interactions. *Oikos*, *121*(10), 1613–1626. https://doi.org/10.1111/j.1600-0706.2011.20085.x

Krofel, M., Giannatos, G., Ćirovič, D., Stoyanov, S., & Newsome, T. M. (2017). Golden jackal expansion in Europe: a case of mesopredator release triggered by continent-wide wolf persecution? *Hystrix, the Italian Journal of Mammalogy*, *28*(1), 9–15. https://doi.org/10.4404/hystrix-28.1-11819

Mazzamuto, M. V., Bisi, F., Wauters, L. A., Preatoni, D. G., & Martinoli, A. (2017). Interspecific competition between alien Pallas’s squirrels and Eurasian red squirrels reduces density of the native species. *Biological Invasions*, *19*(2), 723–735. https://doi.org/10.1007/s10530-016-1310-3

Mitchell-Jones, A. J., Amori, G., Bogdanowicz, W., Krystufek, B., Reijnders, P. J. H., Spitzenberger, F., Stubbe, M., Thissen, J. B. M., Vohralik, V., & Zima, J. (1999). *The Atlas of European Mammals*. Academic Press.

Molinari, P., Rotelli, L., Catello, M., & Bassano, B. (2001). Present status and distribution of the Eurasian lynx (Lynx lynx) in the Italian Alps. *Hystrix*, *12*(2), 3–9.

Trucchi, E., & Sbordoni, V. (2009). Unveiling an ancient biological invasion: molecular analysis of an old European alien, the crested porcupine (Hystrix cristata). *BMC Evolutionary Biology*, *9*(1), 109. https://doi.org/10.1186/1471-2148-9-109

Latvia

Andersone-Lilley, Ž., Balciauskas, L., Ozolinš, J., Randveer, T., & Tõnisson, J. (2010). Ungulates and their management in the Baltics (Estonia, Latvia and Lithuania). In M. Apollonio, R. Andersen, & R. Putman (Eds.), *European ungulates and their management in the 21st century* (pp. 103–128). Cambridge University Press.

Genovesi, P., Bacher, S., Kobelt, M., Pascal, M., & Scalera, R. (2009). Alien Mammals of Europe. In *Handbook of Alien Species in Europe*. Springer.

Halley, D. J., Saveljev, A. P., & Rosell, F. (2021). Population and distribution of beavers *Castor fiber* and *Castor canadensis* in Eurasia. *Mammal Review*, *51*(1), 1–24. https://doi.org/10.1111/mam.12216

Maran, T. (2007). *Conservation biology of the European mink, Mustela lutreola (Linnaeus 1761): decline and causes of extinction*. Tallinn University.

Pagad, S. (2020). Global Register of Introduced and Invasive Species- Latvia. v1.1. *Invasive Species Specialist Group ISSG,* Dataset/Checklist https://cloud.gbif.org/griis/resource?r=griis-latvia&v=1.1 Downloaded via GBIF.org 03/2020.

Timm, U., Pilats, V., & Balciauskas, L. (1998). Mammals of the East Baltic. *Proceedings of the Latvian Academy of Sciences*, *52*(1/2), 1–9.

Liechtenstein

Brambilla, A., Von Hardenberg, A., Nelli, L., & Bassano, B. (2020). Distribution, status, and recent population dynamics of Alpine ibex Capra ibex in Europe. *Mammal Review*, *50*(3), 267–277. https://doi.org/https://doi.org/10.1111/mam.12194

Fasel, M. (2001). The lynx in Liechtenstein. *Hystrix, the Italian Journal of Mammalogy*, *12*(2).

Genovesi, P., Bacher, S., Kobelt, M., Pascal, M., & Scalera, R. (2009). Alien Mammals of Europe. In *Handbook of Alien Species in Europe*. Springer.

Halley, D. J., Saveljev, A. P., & Rosell, F. (2021). Population and distribution of beavers *Castor fiber* and *Castor canadensis* in Eurasia. *Mammal Review*, *51*(1), 1–24. https://doi.org/10.1111/mam.12216

Mueller, O., Wong, L. J., & Pagad, S. (2020). Global Register of Introduced and Invasive Species- Liechtenstein. v1.1. *Invasive Species Specialist Group ISSG,* Dataset/Checklist https://cloud.gbif.org/griis/resource?r=griis-liechtenstein&v=1.1 Downloaded via GBIF.org 03/2020.

Lithuania

Andersone-Lilley, Ž., Balciauskas, L., Ozolinš, J., Randveer, T., & Tõnisson, J. (2010). Ungulates and their management in the Baltics (Estonia, Latvia and Lithuania). In M. Apollonio, R. Andersen, & R. Putman (Eds.), *European ungulates and their management in the 21st century* (pp. 103–128). Cambridge University Press.

Balciauskas, L. (1996). Lithuanian mammal fauna review. *Hystrix, the Italian Journal of Mammalogy*, *8*(2), 9–15. https://doi.org/10.4404/hystrix-8.1-2-4087

Balčiauskas, L. (1999). European Bison (*Bison Bonasus*) in Lithuania: Status and Possibilities of Range Extension. *Acta Zoologica Lituanica*, *9*(3), 3–18. https://doi.org/10.1080/13921657.1999.10512295

Bertolino, S. (2017). Distribution and status of the declining garden dormouse *Eliomys quercinus*. *Mammal Review*, *47*(2), 133–147. https://doi.org/10.1111/mam.12087

Genovesi, P., Bacher, S., Kobelt, M., Pascal, M., & Scalera, R. (2009). Alien Mammals of Europe. In *Handbook of Alien Species in Europe*. Springer.

Halley, D. J., Saveljev, A. P., & Rosell, F. (2021). Population and distribution of beavers *Castor fiber* and *Castor canadensis* in Eurasia. *Mammal Review*, *51*(1), 1–24. https://doi.org/10.1111/mam.12216

Maran, T. (2007). *Conservation biology of the European mink, Mustela lutreola (Linnaeus 1761): decline and causes of extinction*. Tallinn University.

Pagad, S. (2020). Global Register of Introduced and Invasive Species- Lithuania. v1.1. *Invasive Species Specialist Group ISSG,* Dataset/Checklist https://cloud.gbif.org/griis/resource?r=griis-lithuania&v=1.1 Downloaded via GBIF.org 03/2020.

Luxembourg

Cellina, S., & Schley, L. (2014). Premières observations du cerf sika (*Cervus nippon*) au Luxembourg. *Bulletin de la Société des Naturalistes Luxembourgeois*, *115*, 193–194.

Conroy, J. W. H., & Chanin, P. R. F. (2000). The status of the Eurasian otter (*Lutra lutra*) in Europe. A Review. *Journal of the International Otter Survival Fund*, *1*, 7–28.

Genovesi, P., Bacher, S., Kobelt, M., Pascal, M., & Scalera, R. (2009). Alien Mammals of Europe. In *Handbook of Alien Species in Europe*. Springer.

Halley, D. J., Saveljev, A. P., & Rosell, F. (2021). Population and distribution of beavers *Castor fiber* and *Castor canadensis* in Eurasia. *Mammal Review*, *51*(1), 1–24. https://doi.org/10.1111/mam.12216

Ries, C., & Pagad, S. (2020). Global Register of Introduced and Invasive Species GRIIS- Luxembourg. v1.1. *Invasive Species Specialist Group ISSG*, Dataset/Checklist https://cloud.gbif.org/griis/resource?r=griis-luxembourg&v=1.1 Downloaded via GBIF.org 03/2020.

Schley, L., Krier, A., Baghli, A., & Roper, T. J. (1998). Hunting records of game species in Luxembourg during the period 1946 to 1995. *Bulletin de la Société des Naturalistes Luxembourgeois*, *99*, 69–75.

Malta

Cucchi, T., & Vigne, J.-D. (2006). Origin and Diffusion of the House Mouse in the Mediterranean. *Human Evolution*, *21*(2), 95. https://doi.org/10.1007/s11598-006-9011-z

Dobson, M. (1998). Mammal distributions in the western Mediterranean: the role of human intervention. *Mammal Review*, *28*(2), 77–88. https://doi.org/10.1046/j.1365-2907.1998.00027.x

Mifsud, S., Evans, J., Schembri, P. J., Wong, L. J., & Pagad, S. (2020). Global Register of Introduced and Invasive Species- Malta. v1.1. *Invasive Species Specialist Group ISSG,* Dataset/Checklist https://cloud.gbif.org/griis/resource?r=griis_malta&v=1.1 Downloaded via GBIF.org 03/2020.

Rodrigues, M., Bos, A. R., Schembri, P. J., de Lima, R. F., Lymberakis, P., Parpal, L., Cento, M., Ruette, S., Ozkurt, S. O., Santos-Reis, M., Merilä, J., & Fernandes, C. (2017). Origin and introduction history of the least weasel (Mustela nivalis) on Mediterranean and Atlantic islands inferred from genetic data. *Biological Invasions*, *19*(1), 399–421. https://doi.org/10.1007/s10530-016-1287-y

Moldova

Arnold, J., Humer, A., Heltai, M., Murariu, D., Spassov, N., & Hackländer, K. (2012). Current status and distribution of golden jackals Canis aureus in Europe. *Mammal Review*, *42*(1), 1–11. https://doi.org/10.1111/j.1365-2907.2011.00185.x

Crees, J. J., & Turvey, S. T. (2014). Holocene extinction dynamics of *Equus hydruntinus*, a late-surviving European megafaunal mammal. *Quaternary Science Reviews*, *91*, 16–29. https://doi.org/10.1016/j.quascirev.2014.03.003

Genovesi, P., Bacher, S., Kobelt, M., Pascal, M., & Scalera, R. (2009). Alien Mammals of Europe. In *Handbook of Alien Species in Europe*. Springer.

Krofel, M., Giannatos, G., Ćirovič, D., Stoyanov, S., & Newsome, T. M. (2017). Golden jackal expansion in Europe: a case of mesopredator release triggered by continent-wide wolf persecution? *Hystrix, the Italian Journal of Mammalogy*, *28*(1), 9–15. https://doi.org/10.4404/hystrix-28.1-11819

Maran, T. (2007). *Conservation biology of the European mink, Mustela lutreola (Linnaeus 1761): decline and causes of extinction*. Tallinn University.

Nistreanu, V., Munjiu, O., Busmachiu, G., Dumitru, B., Wong, L.J., & Pagad, S. (2020). Global Register of Introduced and Invasive Specie- Moldova. v1.0. *Invasive Species Specialist Group ISSG,* Dataset/Checklist https://cloud.gbif.org/griis/resource?r=griis-moldova&v=1.0 Downloaded via GBIF.org 03/2020.

Montenegro

Arnold, J., Humer, A., Heltai, M., Murariu, D., Spassov, N., & Hackländer, K. (2012). Current status and distribution of golden jackals Canis aureus in Europe. *Mammal Review*, *42*(1), 1–11. https://doi.org/10.1111/j.1365-2907.2011.00185.x

Breitenmoser-Wuersten, C., & Breitenmoser, U. (2001). The lynx in the Balkans - a summary of present knowledge. *KORA Bericht*, *7*, 32–35.

Halley, D. J., & Rosell, F. (2002). The beaver’s reconquest of Eurasia: status, population development and management of a conservation success. *Mammal Review*, *32*(3), 153–178.

Halley, D. J., Saveljev, A. P., & Rosell, F. (2021). Population and distribution of beavers *Castor fiber* and *Castor canadensis* in Eurasia. *Mammal Review*, *51*(1), 1–24. https://doi.org/10.1111/mam.12216

Krofel, M., Giannatos, G., Ćirovič, D., Stoyanov, S., & Newsome, T. M. (2017). Golden jackal expansion in Europe: a case of mesopredator release triggered by continent-wide wolf persecution? *Hystrix, the Italian Journal of Mammalogy*, *28*(1), 9–15. https://doi.org/10.4404/hystrix-28.1-11819

Maran, T. (2007). *Conservation biology of the European mink, Mustela lutreola (Linnaeus 1761): decline and causes of extinction*. Tallinn University.

Mitchell-Jones, A. J., Amori, G., Bogdanowicz, W., Krystufek, B., Reijnders, P. J. H., Spitzenberger, F., Stubbe, M., Thissen, J. B. M., Vohralik, V., & Zima, J. (1999). *The Atlas of European Mammals*. Academic Press.

Pagad, S. (2020). Global Register of Introduced and Invasive Species- Montenegro. v1.1. *Invasive Species Specialist Group ISSG,* Dataset/Checklist https://cloud.gbif.org/griis/resource?r=griis-montenegro&v=1.1 Downloaded via GBIF.org 03/2020.

Netherlands

Genovesi, P., Bacher, S., Kobelt, M., Pascal, M., & Scalera, R. (2009). Alien Mammals of Europe. In *Handbook of Alien Species in Europe*. Springer.

Halley, D. J., Saveljev, A. P., & Rosell, F. (2021). Population and distribution of beavers *Castor fiber* and *Castor canadensis* in Eurasia. *Mammal Review*, *51*(1), 1–24. https://doi.org/10.1111/mam.12216

Maran, T. (2007). *Conservation biology of the European mink, Mustela lutreola (Linnaeus 1761): decline and causes of extinction*. Tallinn University.

Pagad, S., & Wong, L.J. (2020). Global Register of Introduced and Invasive Species – Netherlands. v1.0. *Invasive Species Specialist Group ISSG,* Dataset/Checklist https://cloud.gbif.org/griis/resource?r=griis-nl&v=1.0 Downloaded via GBIF.org 04/2020.

Thissen, J., & Hollander, H. (1996). Status and distribution of mammals in the Netherlands since 1800. *Hystrix-the Italian Journal of Mammalogy*, *8*(1–2).

North Macedonia

Arnold, J., Humer, A., Heltai, M., Murariu, D., Spassov, N., & Hackländer, K. (2012). Current status and distribution of golden jackals Canis aureus in Europe. *Mammal Review*, *42*(1), 1–11. https://doi.org/10.1111/j.1365-2907.2011.00185.x

Breitenmoser-Wuersten, C., & Breitenmoser, U. (2001). The lynx in the Balkans - a summary of present knowledge. *KORA Bericht*, *7*, 32–35.

Genovesi, P., Bacher, S., Kobelt, M., Pascal, M., & Scalera, R. (2009). Alien Mammals of Europe. In *Handbook of Alien Species in Europe*. Springer.

Krofel, M., Giannatos, G., Ćirovič, D., Stoyanov, S., & Newsome, T. M. (2017). Golden jackal expansion in Europe: a case of mesopredator release triggered by continent-wide wolf persecution? *Hystrix, the Italian Journal of Mammalogy*, *28*(1), 9–15. https://doi.org/10.4404/hystrix-28.1-11819

Purger, J., & Kryštufek, B. (1991). Feral Coypu *Myocastor coypus* (Rodentia, Mammalia) in Yugoslavia. *BIOLOSKI VESTNIK*, *39*(4), 19–24.

Trajanovski, S., Simovski, B., Nikolov, B., Wong, L. J., & Pagad, S. (2020). Global Register of Introduced and Invasive Species- North Macedonia. v1.2. *Invasive Species Specialist Group ISSG*, Dataset/Checklist https://cloud.gbif.org/griis/resource?r=griis-macedonia&v=1.2 Downloaded via GBIF.org 03/2020.

Norway

Gederaas, L., Moen, T. L., Skjelseth, S., & Larsen, L.-K. (Eds.). (2012). *Alien species in Norway – with the Norwegian Black List*. The Norwegian Biodiversity Information Centre, Norway.

Genovesi, P., Bacher, S., Kobelt, M., Pascal, M., & Scalera, R. (2009). Alien Mammals of Europe. In *Handbook of Alien Species in Europe*. Springer.

Hilmo, O., Gederaas, L., Wong, L.J., & Pagad, S. (2020). Global Register of Introduced and Invasive Species- Norway. v1.12. *Invasive Species Specialist Group ISSG*, Dataset/Checklist https://cloud.gbif.org/griis/resource?r=griis-brunei-norway&v=1.12 Downloaded via GBIF.org 03/2020.

Lorenzen, E. D., Nogués-Bravo, D., Orlando, L., Weinstock, J., Binladen, J., Marske, K. A., Ugan, A., Borregaard, M. K., Gilbert, M. T. P., Nielsen, R., Ho, S. Y. W., Goebel, T., Graf, K. E., Byers, D., Stenderup, J. T., Rasmussen, M., Campos, P. F., Leonard, J. A., Koepfli, K.-P., … Willerslev, E. (2011). Species-specific responses of Late Quaternary megafauna to climate and humans. *Nature*, *479*(7373), 359–364. https://doi.org/10.1038/nature10574

Råberg, L., Loman, J., Hellgren, O., van der Kooij, J., Isaksen, K., & Solheim, R. (2013). The origin of Swedish and Norwegian populations of the Eurasian harvest mouse (*Micromys minutus*). *Acta Theriologica*, *58*(1), 101–104. https://doi.org/10.1007/s13364-012-0102-0

Poland

Carter, J., & Leonard, B. (2002). A Review of the Literature on the Worldwide Distribution, Spread of, and Efforts to Eradicate the Coypu (Myocastor coypus). *Wildlife Society Bulletin*, *30*(1), 162–175.

Genovesi, P., Bacher, S., Kobelt, M., Pascal, M., & Scalera, R. (2009). Alien Mammals of Europe. In *Handbook of Alien Species in Europe*. Springer.

Halley, D. J., Saveljev, A. P., & Rosell, F. (2021). Population and distribution of beavers *Castor fiber* and *Castor canadensis* in Eurasia. *Mammal Review*, *51*(1), 1–24. https://doi.org/10.1111/mam.12216

Kowalczyk, R., Krasińska, M., Kamiński, T., Górny, M., Struś, P., Hofman-Kamińska, E., & Krasiński, Z. A. (2013). Movements of European bison (*Bison bonasus*) beyond the Białowieża Forest (NE Poland): range expansion or partial migrations? *Acta Theriologica*, *58*(4), 391–401.

Maran, T. (2007). *Conservation biology of the European mink, Mustela lutreola (Linnaeus 1761): decline and causes of extinction*. Tallinn University.

Matějů, J., Říčanová, Š., Ambros, M., Kala, B., Hapl, E., & Matějů, K. (2010). Reintroductions of the European Ground Squirrel (*Spermophilus citellus*) in Central Europe (Rodentia: Sciuridae). *Lynx, Series Nova*, *41*(1).

Solarz, W., Tokarska-Guzik, B., Dajdok, Z., Wong, L.J., & Pagad, S. (2020). Global Register of Introduced and Invasive Species- Poland. v1.1. *Invasive Species Specialist Group ISSG,* Dataset/Checklist https://cloud.gbif.org/griis/resource?r=griis-poland&v=1.1 Downloaded via GBIF.org 03/2020.

Spassov, N. (2009). The south-westernmost distribution of the saiga in the Holocene of Europe: a *Saiga tatarica* find from an archaeological site in Bulgaria. *Saxa Loquuntur. Volume in Honor of the 65th Anniversary of Nikolai Sirakov. Avalon Publisher, Sofia*, 317–322.

Portugal

Bencatel, J., Ferreira, C. C., Barbosa, A. M., Rosalino, L. M., & Álvares, F. (2018). Research trends and geographical distribution of mammalian carnivores in Portugal (SW Europe). *PLOS ONE*, *13*(11), e0207866. https://doi.org/10.1371/journal.pone.0207866

Carvalho, J., Torres, R. T., Acevedo, P., Santos, J. P. V, Barros, T., Serrano, E., & Fonseca, C. (2018). Propagule pressure and land cover changes as main drivers of red and roe deer expansion in mainland Portugal. *Diversity and Distributions*, *24*(4), 551–564. https://doi.org/10.1111/ddi.12703

Crees, J. J., & Turvey, S. T. (2014). Holocene extinction dynamics of *Equus hydruntinus*, a late-surviving European megafaunal mammal. *Quaternary Science Reviews*, *91*, 16–29. https://doi.org/10.1016/j.quascirev.2014.03.003

Genovesi, P., Bacher, S., Kobelt, M., Pascal, M., & Scalera, R. (2009). Alien Mammals of Europe. In *Handbook of Alien Species in Europe*. Springer.

Marchante, H., Marchante, E., Paiva, M., Chainho, P., Anastácio, P., Pinto da Silva Menezes de Sequeira, M., Ribeiro, F., Pires Bento Silva Elias, R. M., Figueiredo, A., Jardim, R., Wong L. J., & Pagad, S. (2020). Global Register of Introduced and Invasive Species- Portugal. v1.5. *Invasive Species Specialist Group ISSG*, Dataset/Checklist https://cloud.gbif.org/griis/resource?r=griis-portugal&v=1.5 Downloaded via GBIF.org 03/2020.

Romania

Arnold, J., Humer, A., Heltai, M., Murariu, D., Spassov, N., & Hackländer, K. (2012). Current status and distribution of golden jackals Canis aureus in Europe. *Mammal Review*, *42*(1), 1–11. https://doi.org/10.1111/j.1365-2907.2011.00185.x

Breitenmoser-Wuersten, C., & Breitenmoser, U. (2001). The lynx in the Balkans - a summary of present knowledge. *KORA Bericht*, *7*, 32–35.

Cogălniceanu, D., Skolka, M., Stanescu, F., Tudor, M., Memedemin, D., Cristina, P., Wong, L. J., & Pagad, S. (2020). Global Register of Introduced and Invasive Species- Romania. v1.1*. Invasive Species Specialist Group ISSG,* Dataset/Checklist https://cloud.gbif.org/griis/resource?r=griis-romania&v=1.1 Downloaded via GBIF.org 03/2020.

Crees, J. J., & Turvey, S. T. (2014). Holocene extinction dynamics of *Equus hydruntinus*, a late-surviving European megafaunal mammal. *Quaternary Science Reviews*, *91*, 16–29. https://doi.org/10.1016/j.quascirev.2014.03.003

Geacu, S. (2006). Mammalian species introduced in some counties of Romania. *The Annals of Valahia University of Târgovişte Geographical Series*, *Tome 6*-*7*, 85–89.

Genovesi, P., Bacher, S., Kobelt, M., Pascal, M., & Scalera, R. (2009). Alien Mammals of Europe. In *Handbook of Alien Species in Europe*. Springer.

Halley, D. J., Saveljev, A. P., & Rosell, F. (2021). Population and distribution of beavers *Castor fiber* and *Castor canadensis* in Eurasia. *Mammal Review*, *51*(1), 1–24. https://doi.org/10.1111/mam.12216

Krofel, M., Giannatos, G., Ćirovič, D., Stoyanov, S., & Newsome, T. M. (2017). Golden jackal expansion in Europe: a case of mesopredator release triggered by continent-wide wolf persecution? *Hystrix, the Italian Journal of Mammalogy*, *28*(1), 9–15. https://doi.org/10.4404/hystrix-28.1-11819

Murariu, D., & Chisamera, G. (2004). *Myocastor coypus* Molina, 1782 (Mammalia: Rodentia: Myocastoridae), a new report along the Danube river in Romania. *Travaux Du Meséum National d’Histoire Naturelle “Grigore Antipa*, *46*, 281–287.

Perzanowski, K., & Olech, W. (2013). Restoration of wisent population within the Carpathian eco-region, Europe. In P. S. Soorae (Ed.), *Global Re-introduction Perspectives: 2013. Further case studies from around the globe* (Vol. 190). IUCN/SSC Re-introduction Specialist Group & Environment Agency-ABU DHABI.

Schmölcke, U., & Zachos, F. E. (2005). Holocene distribution and extinction of the moose (Alces alces, Cervidae) in Central Europe. *Mammalian Biology*, *70*(6), 329–344.

San Marino

Genovesi, P., Bacher, S., Kobelt, M., Pascal, M., & Scalera, R. (2009). Alien Mammals of Europe. In *Handbook of Alien Species in Europe*. Springer.

Pagad, S. (2020). Global Register of Introduced and Invasive Species- San Marino. v1.1. *Invasive Species Specialist Group ISSG,* Dataset/Checklist https://cloud.gbif.org/griis/resource?r=griis-san-marino&v=1.1 Downloaded via GBIF.org 03/2020.

Sardinia

Carnevali, L., Marchini, A., Occhipinti, A., Genovesi, P., Wong, L. J, &. Pagad, S. (2020). Global Register of Introduced and Invasive Species- Italy. v1.4. *Invasive Species Specialist Group ISSG*, Dataset/Checklist https://cloud.gbif.org/griis/resource?r=griis_italy&v=1.4 Downloaded via GBIF.org 03/2020.

Cosson, J.-F., Hutterer, R., Libois, R., Sarà, M., Taberlet, P., & Vogel, P. (2005). Phylogeographical footprints of the Strait of Gibraltar and Quaternary climatic fluctuations in the western Mediterranean: a case study with the greater white-toothed shrew, *Crocidura russula* (Mammalia: Soricidae). *Molecular Ecology*, *14*(4), 1151–1162. https://doi.org/10.1111/j.1365-294X.2005.02476.x

Cucchi, T., Vigne, J.-D., & Auffray, J.-C. (2005). First occurrence of the house mouse (*Mus musculus domesticus* Schwarz & Schwarz, 1943) in the Western Mediterranean: a zooarchaeological revision of subfossil occurrences. *Biological Journal of the Linnean Society*, *84*(3), 429–445. https://doi.org/10.1111/j.1095-8312.2005.00445.x

Frati, F., Hartl, G. B., Lovari, S., Delibes, M., & Markov, G. (1998). Quaternary radiation and genetic structure of the red fox *Vulpes vulpes* in the Mediterranean Basin, as revealed by allozymes and mitochondrial DNA. *Journal of Zoology*, *245*(1), 43–51. https://doi.org/10.1111/j.1469-7998.1998.tb00070.x

Louys, J., Braje, T. J., Chang, C.-H., Cosgrove, R., Fitzpatrick, S. M., Fujita, M., Hawkins, S., Ingicco, T., Kawamura, A., MacPhee, R. D. E., McDowell, M. C., Meijer, H. J. M., Piper, P. J., Roberts, P., Simmons, A. H., van den Bergh, G., van der Geer, A., Kealy, S., & O’Connor, S. (2021). No evidence for widespread island extinctions after Pleistocene hominin arrival. *Proceedings of the National Academy of Sciences*, *118*(20), e2023005118. https://doi.org/10.1073/pnas.2023005118

Suchentrunk, F., Ben Slimen, H., Stamatis, C., Sert, H., Scandura, M., Apollonio, M., & Mamuris, Z. (2006). Molecular Approaches Revealing Prehistoric, Historic, or Recent Translocations and Introductions of Hares (Genus *Lepus*) by Humans. *Human Evolution*, *21*(2), 151. https://doi.org/10.1007/s11598-006-9016-7

Vigne, J.-D. (1992). Zooarchaeology and the biogeographical history of the mammals of Corsica and Sardinia since the last ice age. *Mammal Review*, *22*(2), 87–96. https://doi.org/10.1111/j.1365-2907.1992.tb00124.x

Serbia and Kosovo

Arnold, J., Humer, A., Heltai, M., Murariu, D., Spassov, N., & Hackländer, K. (2012). Current status and distribution of golden jackals Canis aureus in Europe. *Mammal Review*, *42*(1), 1–11. https://doi.org/10.1111/j.1365-2907.2011.00185.x

Crees, J. J., & Turvey, S. T. (2014). Holocene extinction dynamics of *Equus hydruntinus*, a late-surviving European megafaunal mammal. *Quaternary Science Reviews*, *91*, 16–29. https://doi.org/10.1016/j.quascirev.2014.03.003

Genovesi, P., Bacher, S., Kobelt, M., Pascal, M., & Scalera, R. (2009). Alien Mammals of Europe. In *Handbook of Alien Species in Europe*. Springer.

Halley, D. J., Saveljev, A. P., & Rosell, F. (2021). Population and distribution of beavers *Castor fiber* and *Castor canadensis* in Eurasia. *Mammal Review*, *51*(1), 1–24. https://doi.org/10.1111/mam.12216

Krofel, M., Giannatos, G., Ćirovič, D., Stoyanov, S., & Newsome, T. M. (2017). Golden jackal expansion in Europe: a case of mesopredator release triggered by continent-wide wolf persecution? *Hystrix, the Italian Journal of Mammalogy*, *28*(1), 9–15. https://doi.org/10.4404/hystrix-28.1-11819

Maran, T. (2007). *Conservation biology of the European mink, Mustela lutreola (Linnaeus 1761): decline and causes of extinction*. Tallinn University.

Rat, M., Anačkov, G., Wong, L.J., Pagad, S. (2020). Global Register of Introduced and Invasive Species- Serbia. v1.1. *Invasive Species Specialist Group ISSG,* Dataset/Checklist https://cloud.gbif.org/griis/resource?r=griis-serbia&v=1.1 Downloaded via GBIF.org 03/2020.

Sicily

Angelici, F., Laurenti, A., & Nappi, A. (2009). A checklist of the mammals of small Italian islands. *Hystrix-the Italian Journal of Mammalogy*, *20*(1).

Bonfiglio, L., Marra, A. C., & Masini, F. (2000). The contribution of Quaternary vertebrates to palaeoenvironmental and palaeoclimatological reconstructions in Sicily. *Geological Society, London, Special Publications*, *181*(1), 171–184. https://doi.org/10.1144/GSL.SP.2000.181.01.16

Carnevali, L., Marchini, A., Occhipinti, A., Genovesi, P., Wong, L. J., Pagad, S. (2020). Global Register of Introduced and Invasive Species- Italy. v1.4. *Invasive Species Specialist Group ISSG,* Dataset/Checklist https://cloud.gbif.org/griis/resource?r=griis_italy&v=1.4 Downloaded via GBIF.org 03/2020.

Crees, J. J., Carbone, C., Sommer, R. S., Benecke, N., & Turvey, S. T. (2016). Millennial-scale faunal record reveals differential resilience of European large mammals to human impacts across the Holocene. *Proceedings of the Royal Society B: Biological Sciences*, *283*(1827), 20152152. https://doi.org/10.1098/rspb.2015.2152

Cucchi, T., Vigne, J.-D., & Auffray, J.-C. (2005). First occurrence of the house mouse (*Mus musculus domesticus* Schwarz & Schwarz, 1943) in the Western Mediterranean: a zooarchaeological revision of subfossil occurrences. *Biological Journal of the Linnean Society*, *84*(3), 429–445. https://doi.org/10.1111/j.1095-8312.2005.00445.x

Mannino, M. A., Catalano, G., Talamo, S., Mannino, G., Di Salvo, R., Schimmenti, V., Lalueza-Fox, C., Messina, A., Petruso, D., Caramelli, D., Richards, M. P., & Sineo, L. (2012). Origin and Diet of the Prehistoric Hunter-Gatherers on the Mediterranean Island of Favignana (Ègadi Islands, Sicily). *PLOS ONE*, *7*(11), e49802. https://doi.org/10.1371/journal.pone.0049802

Masini, F., Petruso, D., Bonfiglio, L., & Mangano, G. (2008). Origination and extinction patterns of mammals in three central Western Mediterranean islands from the Late Miocene to Quaternary. *Quaternary International*, *182*(1), 63–79. https://doi.org/10.1016/j.quaint.2007.09.020

Mattucci, F., Oliveira, R., Bizzarri, L., Vercillo, F., Anile, S., Ragni, B., Lapini, L., Sforzi, A., Alves, P. C., Lyons, L. A., & Randi, E. (2013). Genetic structure of wildcat (*Felis silvestris*) populations in Italy. *Ecology and Evolution*, *3*(8), 2443–2458. https://doi.org/10.1002/ece3.569

Trocchi, V., & Riga, F. (2005). I lagomorfi in Italia. *Linee Guida per La Conservazione e La Gestione. Min. Politiche Agricole e Forestali–Ist. Naz. Fauna Selvatica, Documenti Tecnici*, *25*, 1–128.

Trucchi, E., & Sbordoni, V. (2009). Unveiling an ancient biological invasion: molecular analysis of an old European alien, the crested porcupine (*Hystrix cristata*). *BMC Evolutionary Biology*, *9*(1), 109. https://doi.org/10.1186/1471-2148-9-109

Slovakia

Arnold, J., Humer, A., Heltai, M., Murariu, D., Spassov, N., & Hackländer, K. (2012). Current status and distribution of golden jackals Canis aureus in Europe. *Mammal Review*, *42*(1), 1–11. https://doi.org/10.1111/j.1365-2907.2011.00185.x

Carter, J., & Leonard, B. (2002). A Review of the Literature on the Worldwide Distribution, Spread of, and Efforts to Eradicate the Coypu (*Myocastor coypus*). *Wildlife Society Bulletin*, *30*(1), 162–175.

Genovesi, P., Bacher, S., Kobelt, M., Pascal, M., & Scalera, R. (2009). Alien Mammals of Europe. In *Handbook of Alien Species in Europe*. Springer.

Halley, D. J., Saveljev, A. P., & Rosell, F. (2021). Population and distribution of beavers *Castor fiber* and *Castor canadensis* in Eurasia. *Mammal Review*, *51*(1), 1–24. https://doi.org/10.1111/mam.12216

Krofel, M., Giannatos, G., Ćirovič, D., Stoyanov, S., & Newsome, T. M. (2017). Golden jackal expansion in Europe: a case of mesopredator release triggered by continent-wide wolf persecution? *Hystrix, the Italian Journal of Mammalogy*, *28*(1), 9–15. https://doi.org/10.4404/hystrix-28.1-11819

Maran, T. (2007). *Conservation biology of the European mink, Mustela lutreola (Linnaeus 1761): decline and causes of extinction*. Tallinn University.

Pagad, S. (2020). Global Register of Introduced and Invasive Species- Slovakia. v1.0. *Invasive Species Specialist Group ISSG,* Dataset/Checklist https://cloud.gbif.org/griis/resource?r=griis-slovakia&v=1.0 Downloaded via GBIF.org 03/2020.

Uhrin, M., Hüttmeir, U., Kipson, M., Estók, P., Sachanowicz, K., Bücs, S., Karapandža, B., Paunović, M., Presetnik, P., & Bashta, A. (2016). Status of Savi’s pipistrelle *Hypsugo savii* (Chiroptera) and range expansion in Central and south‐eastern Europe: a review. *Mammal Review*, *46*(1), 1–16.

Slovenia

Arnold, J., Humer, A., Heltai, M., Murariu, D., Spassov, N., & Hackländer, K. (2012). Current status and distribution of golden jackals Canis aureus in Europe. *Mammal Review*, *42*(1), 1–11. https://doi.org/10.1111/j.1365-2907.2011.00185.x

Crees, J. J., & Turvey, S. T. (2014). Holocene extinction dynamics of *Equus hydruntinus*, a late-surviving European megafaunal mammal. *Quaternary Science Reviews*, *91*, 16–29. https://doi.org/10.1016/j.quascirev.2014.03.003

De Groot, M., Povz, M., Jernej, J., Vrezec, A., Ogris, N., Kus Veenvliet, J., Wong, L. J., & Pagad, S. (2020). Global Register of Introduced and Invasive Species- Slovenia. v1.1. *Invasive Species Specialist Group ISSG,* Dataset/Checklist https://cloud.gbif.org/griis/resource?r=griis-slovenia&v=1.1 Downloaded via GBIF.org 03/2020.

Genovesi, P., Bacher, S., Kobelt, M., Pascal, M., & Scalera, R. (2009). Alien Mammals of Europe. In *Handbook of Alien Species in Europe*. Springer.

Halley, D. J., Saveljev, A. P., & Rosell, F. (2021). Population and distribution of beavers *Castor fiber* and *Castor canadensis* in Eurasia. *Mammal Review*, *51*(1), 1–24. https://doi.org/10.1111/mam.12216

Krofel, M., Giannatos, G., Ćirovič, D., Stoyanov, S., & Newsome, T. M. (2017). Golden jackal expansion in Europe: a case of mesopredator release triggered by continent-wide wolf persecution? *Hystrix, the Italian Journal of Mammalogy*, *28*(1), 9–15. https://doi.org/10.4404/hystrix-28.1-11819

Purger, J., & Kryštufek, B. (1991). Feral Coypu Myocastor coypus (Rodentia, Mammalia) in Yugoslavia. *BIOLOSKI VESTNIK*, *39*(4), 19–24.

Spain

Barrio, I. C., Herrero, J., Bueno, C. G., López, B. C., Aldezabal, A., Campos-Arceiz, A., & García-González, R. (2013). The successful introduction of the alpine marmot *Marmota marmota* in the Pyrenees, Iberian Peninsula, Western Europe. *Mammal Review*, *43*(2), 142–155. https://doi.org/10.1111/j.1365-2907.2012.00212.x

Crees, J. J., & Turvey, S. T. (2014). Holocene extinction dynamics of *Equus hydruntinus*, a late-surviving European megafaunal mammal. *Quaternary Science Reviews*, *91*, 16–29. https://doi.org/10.1016/j.quascirev.2014.03.003

Dana, E. D., García-Berthou, E., Wong, L. J., & Pagad, S. (2020). Global Register of Introduced and Invasive Species- Spain. v1.1. *Invasive Species Specialist Group ISSG,* Dataset/Checklist https://cloud.gbif.org/griis/resource?r=griis-spain&v=1.1 Downloaded via GBIF.org 03/2020.

Gaubert, P., Machordom, A., Morales, A., López-Bao, J. V., Veron, G., Amin, M., Barros, T., Basuony, M., Djagoun, C. A. M. S., San, E. D. L., Fonseca, C., Geffen, E., Ozkurt, S. O., Cruaud, C., Couloux, A., & Palomares, F. (2011). Comparative phylogeography of two African carnivorans presumably introduced into Europe: disentangling natural versus human-mediated dispersal across the Strait of Gibraltar. *Journal of Biogeography*, *38*(2), 341–358. https://doi.org/10.1111/j.1365-2699.2010.02406.x

Genovesi, P., Bacher, S., Kobelt, M., Pascal, M., & Scalera, R. (2009). Alien Mammals of Europe. In *Handbook of Alien Species in Europe*. Springer.

Halley, D. J., & Rosell, F. (2003). *Population and distribution of European beavers (Castor fiber)*. Lutra 46(2), 91-101.

Skyrienė, G., & Paulauskas, A. (2012). Distribution of invasive muskrats (*Ondatra zibethicus*) and impact on ecosystem. *Ekologija*, *58*(3).

Sweden

Genovesi, P., Bacher, S., Kobelt, M., Pascal, M., & Scalera, R. (2009). Alien Mammals of Europe. In *Handbook of Alien Species in Europe*. Springer.

Halley, D. J., Saveljev, A. P., & Rosell, F. (2021). Population and distribution of beavers *Castor fiber* and *Castor canadensis* in Eurasia. *Mammal Review*, *51*(1), 1–24. https://doi.org/10.1111/mam.12216

Hofman-Kamińska, E., Bocherens, H., Drucker, D. G., Fyfe, R. M., Gumiński, W., Makowiecki, D., Pacher, M., Piličiauskienė, G., Samojlik, T., Woodbridge, J., & Kowalczyk, R. (2019). Adapt or die—Response of large herbivores to environmental changes in Europe during the Holocene. *Global Change Biology*, *25*(9), 2915–2930. https://doi.org/10.1111/gcb.14733

Lorenzen, E. D., Nogués-Bravo, D., Orlando, L., Weinstock, J., Binladen, J., Marske, K. A., Ugan, A., Borregaard, M. K., Gilbert, M. T. P., Nielsen, R., Ho, S. Y. W., Goebel, T., Graf, K. E., Byers, D., Stenderup, J. T., Rasmussen, M., Campos, P. F., Leonard, J. A., Koepfli, K.-P., … Willerslev, E. (2011). Species-specific responses of Late Quaternary megafauna to climate and humans. *Nature*, *479*(7373), 359–364. https://doi.org/10.1038/nature10574

Pagad, S., & Wong, L. J. (2020). Global Register of Introduced and Invasive Species – Sweden. v1.1. *Invasive Species Specialist Group ISSG,* Dataset/Checklist https://cloud.gbif.org/griis/resource?r=griis-sweden&v=1.1 Downloaded via GBIF.org 04/2020.

Truvé, J., & Lemel, J. (2003). Timing and distance of natal dispersal for wild boar *Sus scrofa* in Sweden. *Wildlife Biology*, *9*(4), 51–57. https://doi.org/10.2981/wlb.2003.056

Wabakken, P., Sand, H., Liberg, O., & Bjärvall, A. (2001). The recovery, distribution, and population dynamics of wolves on the Scandinavian peninsula, 1978-1998. *Canadian Journal of Zoology*, *79*(4), 710–725. https://doi.org/10.1139/z01-029

Switzerland

Arlettaz, R., Godat, S., & Meyer, H. (2000). Competition for food by expanding pipistrelle bat populations (*Pipistrellus pipistrellus*) might contribute to the decline of lesser horseshoe bats (*Rhinolophus hipposideros*). *Biological Conservation*, *93*(1), 55–60.

Genovesi, P., Bacher, S., Kobelt, M., Pascal, M., & Scalera, R. (2009). Alien Mammals of Europe. In *Handbook of Alien Species in Europe*. Springer.

Maran, T. (2007). *Conservation biology of the European mink, Mustela lutreola (Linnaeus 1761): decline and causes of extinction*. Tallinn University.

Nolet, B. A., & Rosell, F. (1998). Comeback of the beaver *Castor fiber*: An overview of old and new conservation problems. *Biological Conservation*, *83*(2), 165–173. https://doi.org/10.1016/S0006-3207(97)00066-9

Pesaresi, J., & Ruedi, M. (2020). First record of a presumed wild common genet (*Genetta genetta*) in Switzerland. *Revue Suisse de Zoologie*, *127*(1), 101–104. https://doi.org/10.35929/RSZ.0010

Stüwe, M., & Nievergelt, B. (1991). Recovery of alpine ibex from near extinction: the result of effective protection, captive breeding, and reintroductions. *Applied Animal Behaviour Science*, *29*(1–4), 379–387.

Weber, E., Wong, L.J., & Pagad, S. (2020). Global Register of Introduced and Invasive Species- Switzerland. v1.1. *Invasive Species Specialist Group ISSG*, Dataset/Checklist https://cloud.gbif.org/griis/resource?r=griis-switzerland&v=1.1 Downloaded via GBIF.org 03/2020.

Ukraine

Aleksandrov, B., Wong, L. J., & Pagad, S. (2020). Global Register of Introduced and Invasive Species- Ukraine. v1.1. *Invasive Species Specialist Group ISSG,* Dataset/Checklist https://cloud.gbif.org/griis/resource?r=griis-ukraine&v=1.1 Downloaded via GBIF.org 03/2020.

Arnold, J., Humer, A., Heltai, M., Murariu, D., Spassov, N., & Hackländer, K. (2012). Current status and distribution of golden jackals Canis aureus in Europe. *Mammal Review*, *42*(1), 1–11. https://doi.org/10.1111/j.1365-2907.2011.00185.x

Carter, J., & Leonard, B. (2002). A Review of the Literature on the Worldwide Distribution, Spread of, and Efforts to Eradicate the Coypu (Myocastor coypus). *Wildlife Society Bulletin*, *30*(1), 162–175.

Crees, J. J., & Turvey, S. T. (2014). Holocene extinction dynamics of *Equus hydruntinus*, a late-surviving European megafaunal mammal. *Quaternary Science Reviews*, *91*, 16–29. https://doi.org/10.1016/j.quascirev.2014.03.003

Genovesi, P., Bacher, S., Kobelt, M., Pascal, M., & Scalera, R. (2009). Alien Mammals of Europe. In *Handbook of Alien Species in Europe*. Springer.

Halley, D. J., Saveljev, A. P., & Rosell, F. (2021). Population and distribution of beavers *Castor fiber* and *Castor canadensis* in Eurasia. *Mammal Review*, *51*(1), 1–24. https://doi.org/10.1111/mam.12216

Krofel, M., Giannatos, G., Ćirovič, D., Stoyanov, S., & Newsome, T. M. (2017). Golden jackal expansion in Europe: a case of mesopredator release triggered by continent-wide wolf persecution? *Hystrix, the Italian Journal of Mammalogy*, *28*(1), 9–15. https://doi.org/10.4404/hystrix-28.1-11819

Perzanowski, K., Olech, W., & Kozak, I. (2004). Constraints for re-establishing a meta-population of the European bison in Ukraine. *Biological Conservation*, *120*(3), 345–353. https://doi.org/10.1016/j.biocon.2004.03.006
